# Haploinsufficiency of *CYP8B1* associates with increased insulin sensitivity in humans

**DOI:** 10.1172/JCI152961

**Published:** 2022-11-01

**Authors:** Shiqi Zhong, Raphael Chèvre, David Castaño Mayan, Maria Corlianò, Blake J. Cochran, Kai Ping Sem, Theo H. van Dijk, Jianhe Peng, Liang Juin Tan, Siddesh V. Hartimath, Boominathan Ramasamy, Peter Cheng, Albert K. Groen, Folkert Kuipers, Julian L. Goggi, Chester Drum, Rob M. van Dam, Ru San Tan, Kerry-Anne Rye, Michael R. Hayden, Ching-Yu Cheng, Shaji Chacko, Jason Flannick, Xueling Sim, Hong Chang Tan, Roshni R. Singaraja

**Affiliations:** 1 Department of Medicine, Yong Loo Lin School of Medicine, National University of Singapore, Singapore.; 2 Translational Laboratory in Genetic Medicine, Agency for Science, Technology and Research (A*STAR), Singapore.; 3 Cardiovascular Research Institute, National University Health System, Singapore.; 4 School of Medical Sciences, University of New South Wales, Sydney, Australia.; 5 Departments of Pediatrics and Laboratory Medicine, University of Groningen, University Medical Center Groningen, Netherlands.; 6 Experimental Therapeutics Centre and; 7 Singapore Bioimaging Consortium, A*STAR, Singapore.; 8 Saw Swee Hock School of Public Health, National University of Singapore and National University Health System, Singapore.; 9 Department of Cardiology, National Heart Centre, Singapore.; 10 Department of Medical Genetics, Centre for Molecular Medicine and Therapeutics, The University of British Columbia, Vancouver, British Columbia, Canada.; 11 Singapore Eye Research Institute, Singapore National Eye Centre, Singapore.; 12 Ophthalmology and Visual Sciences Academic Clinical Program (Eye ACP), Duke-NUS Medical School, Singapore.; 13 USDA/ARS Children’s Nutrition Research Centre, Department of Pediatrics, Baylor College of Medicine, Houston, Texas, USA.; 14 Program in Metabolism and; 15 Program in Medical & Population Genetics, Broad Institute, Cambridge, Massachusetts, USA.; 16 Division of Genetics and Genomics, Boston Children’s Hospital, Boston, Massachusetts, USA.; 17 Department of Pediatrics, Harvard Medical School, Boston, Massachusetts, USA.; 18 Department of Endocrinology, Singapore General Hospital, Singapore.

**Keywords:** Endocrinology, Insulin signaling

## Abstract

**BACKGROUND:**

Cytochrome P450 family 8 subfamily B member 1 (CYP8B1) generates 12α-hydroxylated bile acids (BAs) that are associated with insulin resistance in humans.

**METHODS:**

To determine whether reduced CYP8B1 activity improves insulin sensitivity, we sequenced *CYP8B1* in individuals without diabetes and identified carriers of complete loss-of-function (CLOF) mutations utilizing functional assays.

**RESULTS:**

Mutation carriers had lower plasma 12α-hydroxylated/non–12α-hydroxylated BA and cholic acid (CA)/chenodeoxycholic acid (CDCA) ratios compared with age-, sex-, and BMI-matched controls. During insulin clamps, hepatic glucose production was suppressed to a similar magnitude by insulin, but glucose infusion rates to maintain euglycemia were higher in mutation carriers, indicating increased peripheral insulin sensitivity. Consistently, a polymorphic CLOF *CYP8B1* mutation associated with lower fasting insulin in the AMP-T2D-GENES study. Exposure of primary human muscle cells to mutation-carrier CA/CDCA ratios demonstrated increased FOXO1 activity, and upregulation of both insulin signaling and glucose uptake, which were mediated by increased CDCA. Inhibition of FOXO1 attenuated the CDCA-mediated increase in muscle insulin signaling and glucose uptake. We found that reduced CYP8B1 activity associates with increased insulin sensitivity in humans.

**CONCLUSION:**

Our findings suggest that increased circulatory CDCA due to reduced CYP8B1 activity increases skeletal muscle insulin sensitivity, contributing to increased whole-body insulin sensitization.

**FUNDING:**

Biomedical Research Council/National Medical Research Council of Singapore.

## Graphical abstract

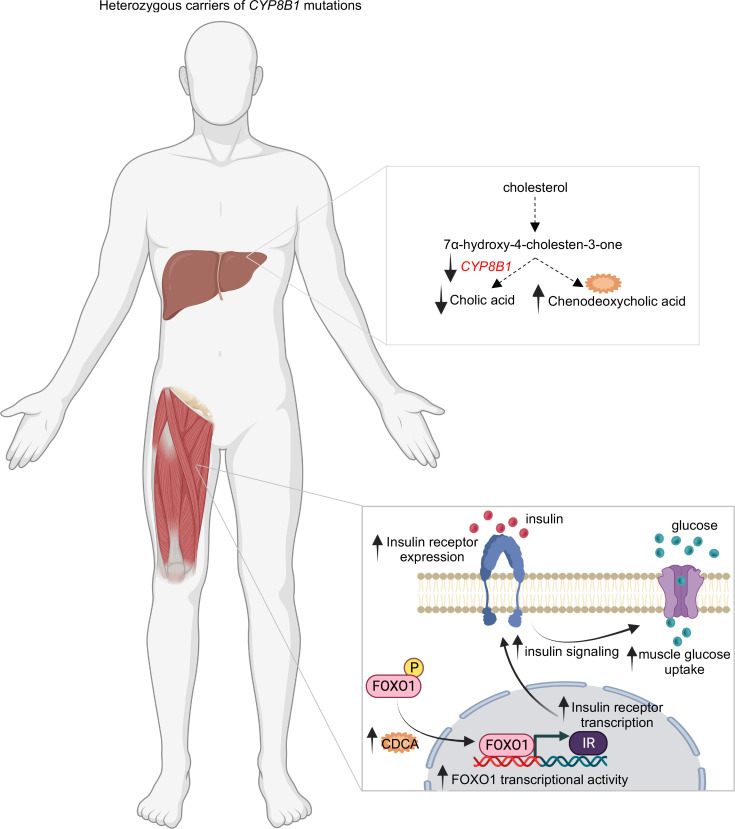

## Introduction

Bile acids (BAs) are products of cholesterol catabolism, accounting for approximately 50% of daily cholesterol turnover in humans, and act as surfactants to promote intestinal lipid absorption (1). The primary products of BA biosynthetic pathways in humans are cholic acid (CA) and chenodeoxycholic acid (CDCA). Intestinal bacteria act on primary BAs to form the secondary BAs deoxycholic acid (DCA) and lithocholic acid (LCA) from CA and CDCA, respectively (1). Two pathways exist for BA synthesis, the neutral and the acidic pathways, with the neutral pathway being predominant in humans. The sterol 12α-hydroxylase CYP8B1 (cytochrome P450, family 8, subfamily B, polypeptide 1) is thought to act in the neutral BA synthesis pathway, catalyzing the 12α-hydroxylation of 7α-hydroxy-4-cholesten-3-one to generate 7α,12α-dihydroxy-4-cholesten-3-one (1). The balance between these 2 steroids determines the relative amounts of CA and CDCA, which in turn determines the hydrophobicity and biological properties of the BA pool. Thus, CYP8B1 is hypothesized to be a critical modulator of BA metabolism in humans. Mice lacking *Cyp8b1* show almost no 12α-hydroxylated BAs and elevated non–12α-hydroxylated BAs (2), suggesting that reduced CYP8B1 activity in humans may result in similar alterations in BA pool composition.

BAs have been recognized as signaling molecules, with different BA species showing distinct signaling properties (1). BAs activate the nuclear farnesoid X receptor (FXR, encoded by *NR1H4*) and membrane G protein–coupled BA receptor (GPBAR1, also known as TGR5) (1), among others. These BA-activated signaling pathways have been implicated in the control of glucose metabolism (1).

There is substantial data linking BAs to obesity and diabetes risk (1). In healthy individuals, insulin resistance is associated with increased plasma 12α-hydroxylated BAs (3), suggesting that increased CYP8B1 activity may associate with insulin resistance. In both nondiabetic and diabetic individuals, a higher 12α-hydroxylated/non–12α-hydroxylated BA ratio correlated with greater insulin resistance (3), again suggesting that increased CYP8B1 activity may contribute to insulin resistance. On the other hand, individuals treated with the FXR agonist obeticholic acid, an analog of the non–12α-hydroxylated BA CDCA, displayed improved insulin sensitivity (4). Collectively, these observations point to a critical role for BAs in the control of glucose metabolism and suggest that decreased CYP8B1 activity in humans may lead to increased insulin sensitivity via a decrease in 12α-hydroxylated and a concomitant increase in non–12α-hydroxylated BAs.

No published studies to our knowledge have identified loss-of-function mutations in *CYP8B1* or assessed the role of CYP8B1 in humans in modulating plasma BA composition and insulin sensitivity. Further, how reduced CYP8B1 activity may affect insulin sensitivity is also unknown. We identify and characterize carriers of loss-of-function mutations in *CYP8B1* and show that reduced CYP8B1 activity increases insulin sensitivity.

## Results

### Complete loss-of-function mutations in CYP8B1 identified in humans.

**Figure 1:**
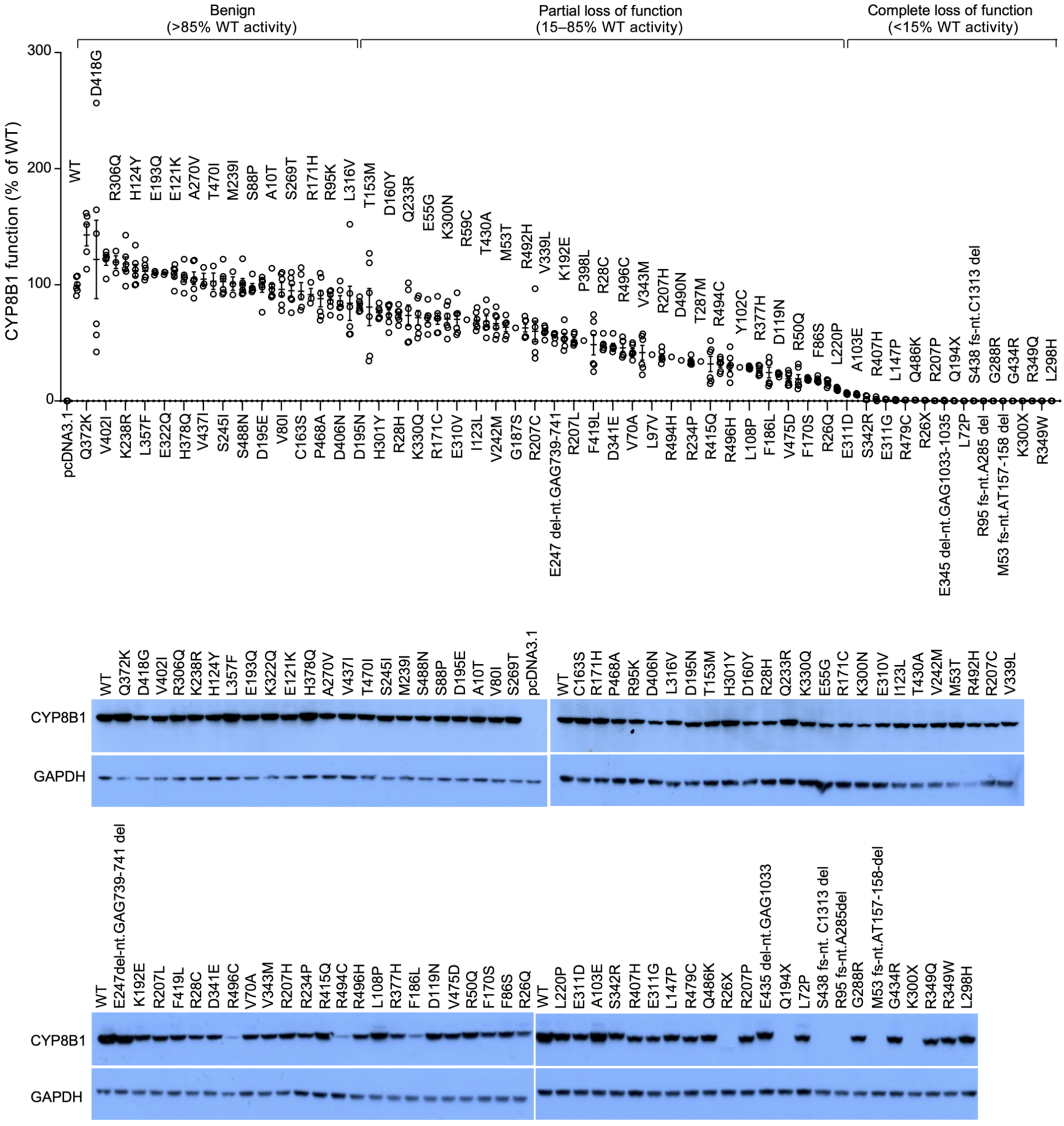
Identification of loss-of-function mutations in *CYP8B1*. *CYP8B1* was Sanger sequenced in 8112 individuals and 100 nonsynonymous variants, frameshifts, and insertions/deletions in the coding region were identified. Each variant was generated by site-directed mutagenesis in human *CYP8B1* cDNA; in vitro assays were performed to quantify the CYP8B1 product 7α,12α-dihydroxy-4-cholesten-3-one for each variant, which was then graphed as a percentage of the substrate generated by wild-type CYP8B1. Twenty-three complete loss-of-function mutations in *CYP8B1* were identified. *n* = 3–6 per variant, with each *n* performed in triplicate. Data are mean ± SEM. See complete unedited blots in the supplemental material.

We sequenced *CYP8B1* in 8112 Singaporean Malay and Chinese individuals, identifying 100 nonsynonymous variants (Supplemental Table 1; supplemental material available online with this article; https://doi.org/10.1172/JCI152961DS1). Of these, 58 were predicted to be possibly or probably damaging by the functional prediction tool Polyphen 2.0 (Polymorphism Phenotyping; http://genetics.bwh.harvard.edu/pph2/), and 41 were predicted to be damaging by SIFT (sorting intolerant from tolerant; http://sift-dna.org). We generated all 100 variants in human *CYP8B1* cDNA, quantified the product generated by each variant, and found a spectrum of defective CYP8B1 activities, classified as complete loss of function (CLOF) (<15% activity of wild-type), partial loss of function (PLOF) (15% to 85% activity of wild-type), or benign (>85% activity of wild-type) (Figure 1 and Supplemental Table 1). We identified 23 CLOF, 50 PLOF, and 27 benign *CYP8B1* variants. A total of 138 individuals carried CLOF, 84 individuals carried PLOF, and 237 individuals carried benign variants. Additionally, 296 individuals carried synonymous variants. To the best of our knowledge these represent the first described human *CYP8B1* mutations. A total of 7357 individuals carried no *CYP8B1* variants, from whom controls were recruited. All carriers were heterozygous. No homozygotes or compound heterozygotes carrying CLOF mutations were identified.

### Baseline characteristics of CYP8B1 mutation carriers and controls.

We recruited 23 heterozygous CLOF *CYP8B1* mutation carriers, and 41 age-, sex-, BMI-, and race-matched controls to our clinical study. Table 1 lists baseline characteristics. Mutation carriers had significantly decreased total cholesterol/HDL-C (*P* = 0.04) and APOB/APOA-I (*P* = 0.04) ratios, suggesting reduced risk for atherosclerosis. In addition, high-sensitivity C-reactive protein (hs-CRP) levels were decreased by approximately 50% in the mutation carriers (*P* = 0.06), suggesting reduced systemic inflammatory status and lower atherosclerotic risk in carriers (5). Although nonsignificant, liver fat was decreased by approximately 30% in carriers, suggesting reduced hepatic steatosis.

### Altered BA pool in heterozygous CYP8B1 mutation carriers.

**Figure 2:**
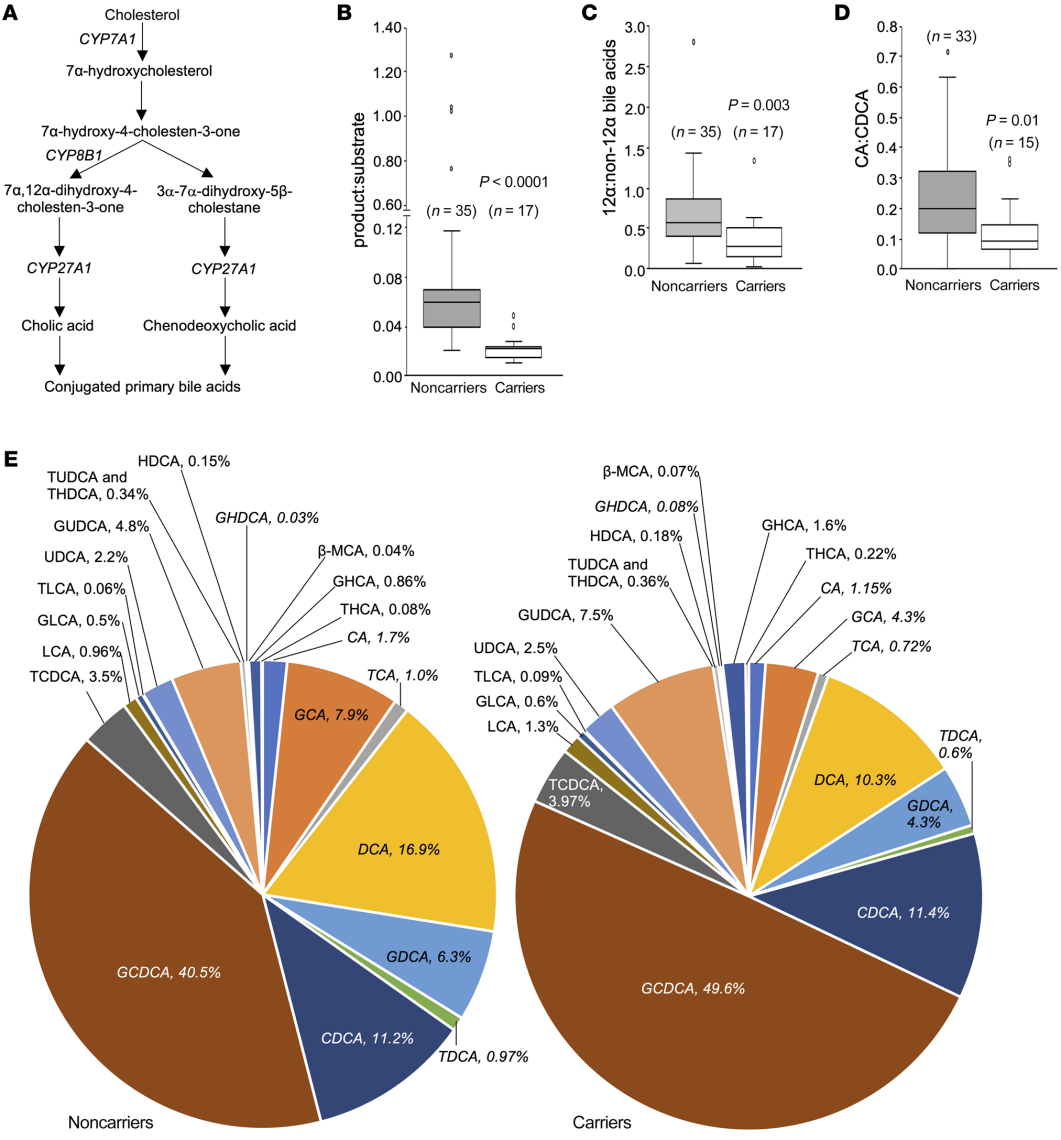
Altered plasma bile acid composition in heterozygous carriers of *CYP8B1* mutations. CYP8B1 is a 12α-hydroxylase, catalyzing the conversion of 7α-hydroxy-4-cholesten-3-one to generate 7α,12α-dihydroxy-4-cholesten-3-one. (**A**) Schematic of the bile acid synthesis pathway with CYP8B1 indicated. (**B**) The ratio of CYP8B1 product (7α,12α-dihydroxy-4-cholesten-3-one) to substrate (7α-hydroxy-4-cholesten-3-one) is decreased in mutation carriers. In addition, the ratios of (**C**) 12α to non-12α bile acids, and (**D**) cholic acid (CA) to chenodeoxycholic acid (CDCA) are decreased in *CYP8B1* mutation carriers. (**E**) The composition of the plasma bile acid pool is shown in carriers and noncarriers. In italic font are the bile acids significantly different between mutation carriers and controls (also shown in Supplemental Table 2). Data (nonparametric) are shown as box-and-whisker plots with median (horizontal lines), interquartile range (boxes), and whiskers generated by Tukey’s method, and were analyzed using the Mann-Whitney *U* test.

*CYP8B1* mutation carriers have not been described so far. Thus, CYP8B1’s role in human BA metabolism has not been directly evaluated. A schematic of the neutral BA synthesis pathway is shown in Figure 2A. No significant differences in the CYP8B1 product 7α,12α-dihydroxy-4-cholesten-3-one were observed in carrier plasma (Supplemental Table 2). However, total downstream 12α-hydroxylated BAs were decreased by 52% (median [IQR] nmol/L: controls, 1449.9 [1442.5]; carriers, 692.5 [577.1]; *P* = 0.0002) (Supplemental Table 2). CYP8B1’s substrate 7α-hydroxy-4-cholesten-3-one was increased (controls, 5.9 [7.4]; carriers, 11.8 [16.4] nmol/mL; *P* = 0.02), resulting in a 63% decrease in the CYP8B1 product/substrate ratio (controls, 0.06 [0.032]; carriers, 0.022 [0.009]; *P* < 0.0001) (Figure 2B and Supplemental Table 2).

The primary BA generated by CYP8B1 is the 12α-hydroxylated bile acid, CA (2). Under conditions of reduced CYP8B1, decreased CA and increased CDCA and its derivatives (non–12α-hydroxylated BAs) are expected (Figure 2A). The 12α-hydroxylated/non–12α-hydroxylated BA ratio was decreased by 49% in mutation carriers (controls, 0.57 [0.46]; carriers, 0.29 [0.37], *P* = 0.003) (Figure 2C). Unconjugated CA was decreased by 42% (controls, 54.5 [59.9]; carriers, 31.8 [17.5] nmol/L; *P* = 0.01), and both glycine- (*P* = 0.01) and taurine-conjugated (*P* = 0.048) CA were decreased (Supplemental Table 2). CDCA did not increase in the carriers. However, the ratio of CDCA and its conjugates (GCDCA and TCDCA) to total BAs (12α-hydroxylated + non–12α-hydroxylated BAs) was increased by 30% in the mutation carriers (controls, 0.46 [0.17]; carriers, 0.60 [0.16]; *P* = 0.03) (Supplemental Table 2). The CA/CDCA ratio was lower in carriers (controls, 0.20 [0.21]; carriers, 0.10 [0.10]; *P* = 0.01) (Figure 2D and Supplemental Table 2). The BA profiles are shown in Figure 2E. Overall, ratios of CYP8B1 product/substrate, 12α-hydroxylated/non–12α-hydroxylated BA, and CA/CDCA were significantly decreased, and the ratio of CDCA to its conjugates total 12α-hydroxylated and non–12α-hydroxylated BAs was significantly increased in *CYP8B1* mutation carriers, despite the fact that they were heterozygous, and likely harbored 50% or greater wild-type CYP8B1 activity.

### Increased peripheral insulin sensitivity in heterozygous CYP8B1 mutation carriers.

**Figure 3:**
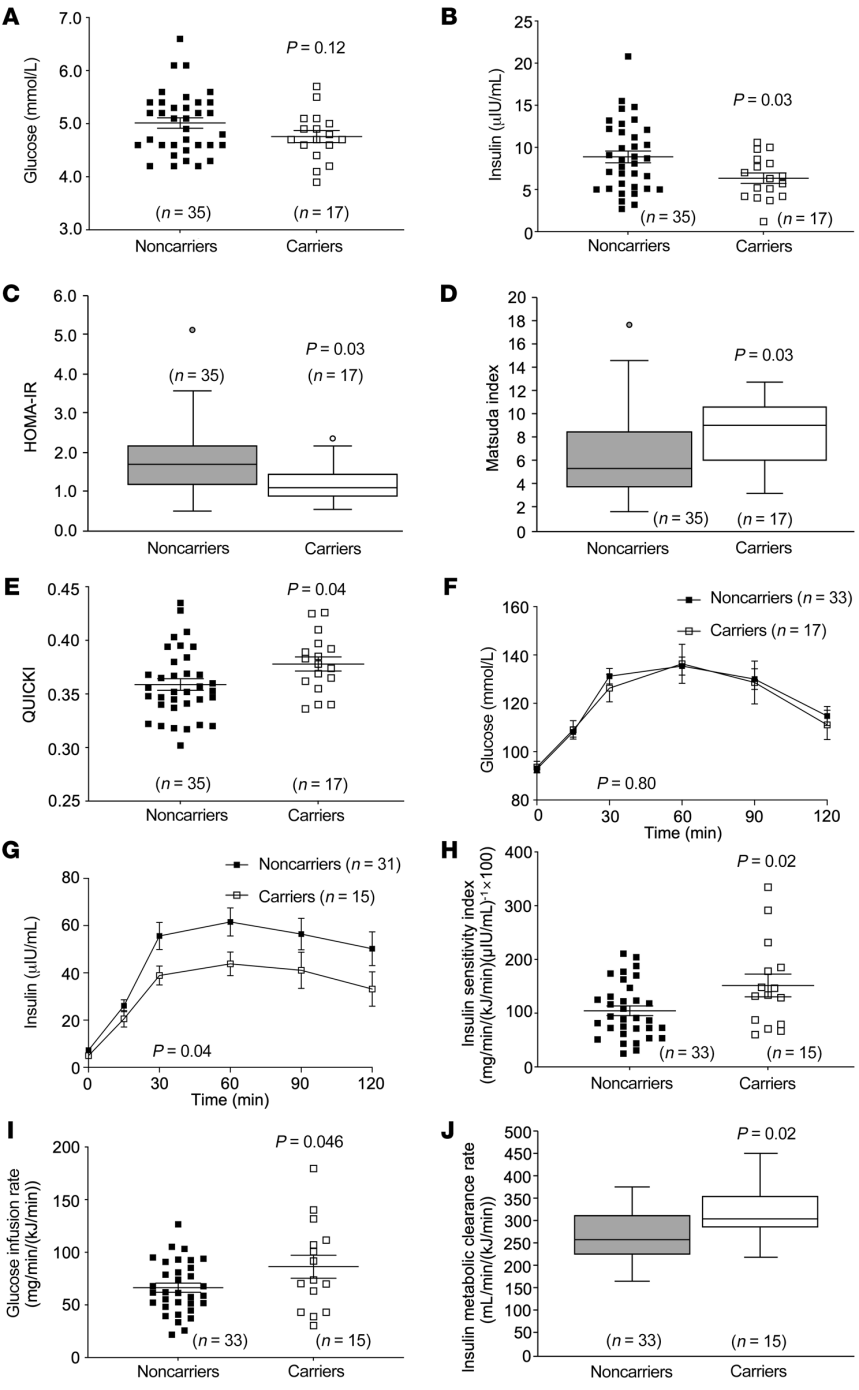
Heterozygous carriers of *CYP8B1* mutations show increased insulin sensitization. (**A**) Fasting plasma glucose was unchanged, and (**B**) fasting plasma insulin levels were decreased in *CYP8B1* mutation carriers. (**C**) Homeostatic model assessment for insulin resistance (HOMA-IR) was decreased, and (**D**) Matsuda Index (a measure of whole-body insulin sensitivity) and (**E**) QUICKI (quantitative insulin-sensitivity check index) were increased in mutation carriers. During mixed-meal tolerance testing, (**F**) plasma glucose levels were unchanged, but (**G**) plasma insulin levels were decreased in mutation carriers. During hyperinsulinemic-euglycemic clamps, (**H**) the insulin sensitivity index, (**I**) glucose infusion rate, and (**J**) insulin metabolic clearance rate were increased. Error bars in **F** and **G** represent SEM. Parametric data in **A**, **B**, **E**, **H**, and **I** are shown as scatter plots with mean ± SEM and were analyzed using unpaired *t* tests. Nonparametric data in **C**, **D**, and **J** are shown as box-and-whisker plots with median (horizontal lines), interquartile range (boxes), and whiskers generated by Tukey’s method, and were analyzed using the Mann-Whitney *U* test. Data in **F** and **G** were analyzed using 2-way ANOVA.

12α-Hydroxylated BAs correlate with insulin resistance in humans (3), and 12α-hydroxylated BAs were decreased in the *CYP8B1* mutation carriers. Thus, we assessed whether *CYP8B1* mutation carriers showed improved insulin sensitivity. Although fasting glucose was unchanged, fasting insulin was decreased by 28% in carriers (controls, 8.9 ± 0.7; carriers, 6.4 ± 0.6 μIU/mL; *P* = 0.03) (Figure 3, A and B). Accordingly, the homeostatic model assessment for insulin resistance (HOMA-IR) (6) was decreased (controls, 1.7 [1.0]; carriers, 1.1 [0.6]; *P* = 0.03) (Figure 3C). The Matsuda Index, which measures whole-body insulin sensitivity (7) (controls, 5.4 [4.6]; carriers, 9.0 [4.5]; *P* = 0.03), as well as QUICKI, another measure of insulin sensitivity (7) (controls, 0.36 ± 0.005; carriers, 0.38 ± 0.007; *P* = 0.04) (Figure 3, D and E), were increased. During mixed-meal tolerance testing (MMTT), although glucose levels were similar in both groups (ANOVA *P* = 0.80, area under the curve [AUC] *P* = 0.65), insulin levels were decreased in carriers (ANOVA *P* = 0.04; AUC: controls, 5877.8 [4543.5]; carriers, 4215.0 [1907.3]; *P* = 0.03) (Figure 3, F and G). During hyperinsulinemic-euglycemic clamps, insulin sensitivity index (ISI) was increased by 45% (controls, 104.4 ± 9.0; carriers, 151.6 ± 21.2 mg/min/[kJ/min] × [mL/μIU] × 100; *P* = 0.02) and the glucose infusion rate (GIR) was increased by 30% in carriers (controls, 66.4 ± 4.3; carriers, 86.4 ± 11.0 mg/min/[kJ/min]; *P* = 0.046) (Figure 3, H and I). The insulin metabolic clearance rate was also increased (controls, 258.2 [82.5]; carriers, 302.1 [68.1] mL/min/[kJ/min]; *P* = 0.02) (Figure 3J). The above measures were normalized to resting energy expenditure at clamp initiation (8). Alternate calculations of ISI and GIR normalized to fat-free mass (8) found ISI to be increased by 33% (controls, 10.7 [7.8]; carriers, 14.2 [7.9] [mg/kg/min] × [mL/μIU] × 100; *P* = 0.04) and GIR by 25% (controls, 7.0 ± 0.4; carriers, 8.8 ± 1.1 mg/kg/min; *P* = 0.07) in carriers.

**Figure 4:**
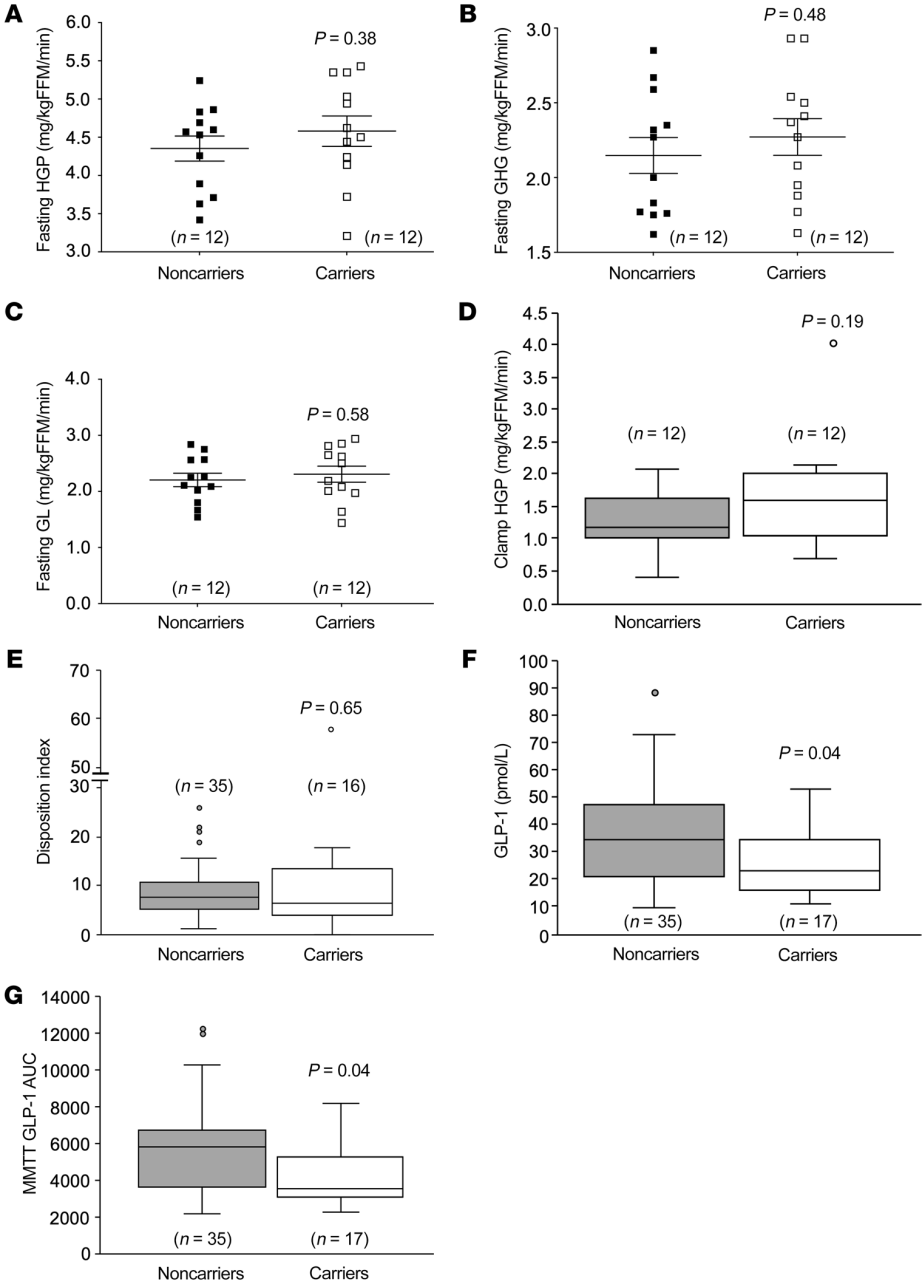
Increased peripheral insulin sensitivity and lower insulin and GLP-1 response during mixed-meal tolerance testing in *CYP8B1* mutation carriers. (**A**) Hepatic glucose production (HGP), (**B**) gluconeogenesis (GNG), and (**C**) glycogenolysis (GL) were unchanged under fasting conditions in the *CYP8B1* mutation carriers. (**D**) During hyperinsulinemic clamps, HGP was unchanged between carriers and controls. However, HGP was equally reduced in carriers and controls during hyperinsulinemic clamps compared to fasted conditions. (**E**) The disposition index, a measure of β cell function, was unchanged, and (**F**) fasting plasma GLP-1, as well as (**G**) GLP-1 levels during mixed-meal tolerance testing (MMTT) were significantly decreased in mutation carriers. FFM, fat-free mass. Parametric data in **A**–**C** are shown as scatter plots with mean ± SEM, and were analyzed using unpaired *t* tests. Nonparametric data in **D**–**G** are shown as box-and-whisker plots with median (horizontal lines), interquartile range (boxes), and whiskers generated by Tukey’s method, and were analyzed using the Mann-Whitney *U* test.

Under fasting conditions, hepatic glucose production (controls, 4.4 ± 0.2; carriers, 4.6 ± 0.2; *P* = 0.38) (Figure 4A), gluconeogenesis (controls, 2.1 ± 0.1; carriers, 2.3 ± 0.1; *P* = 0.48) (Figure 4B), and glycogenolysis (controls, 2.2 ± 0.1; carriers, 2.3 ± 0.1; *P* = 0.58) (Figure 4C) (all mg/kg fat-free mass/min) were not different between carriers and controls. Similarly, during continuous insulin infusion, hepatic glucose production was suppressed to an equal extent in both groups (controls, 1.2 [0.6]; carriers, 1.6 [1.0], mg/kg fat-free mass/min; *P* = 0.19) (Figure 4D), indicating that hepatic insulin sensitivity was not different between the 2 groups. Together, these data suggest that haploinsufficiency of *CYP8B1* significantly increases peripheral insulin sensitivity in humans.

### A polymorphic nonsense mutation in CYP8B1 associates with lower fasting insulin in 45,231 exomes.

To confirm that humans with CLOF *CYP8B1* mutations have improved insulin sensitivity, we performed association analyses using the Type 2 Diabetes Knowledge Portal (http://www.type2diabetesgenetics.org), which enables association analyses between coding variation and glycemic traits in 45,231 exomes (9). Of the 100 nonsynonymous *CYP8B1* variants (Supplemental Table 1), only 1 (*R26X*) was both a CLOF mutation (0.6% activity compared with wild-type CYP8B1), common in our study cohort (1.6% mutation frequency in Malays, Genome Aggregation Database [gnomAD v2.1.1, https://gnomad.broadinstitute.org/] allele frequency = 8.58 × 10^–5^), and present in several copies in the AMP-T2D-GENES data set. Association analyses showed that carriers of *R26X* had significantly lower fasting insulin levels after adjusting for BMI (*P* = 0.02; effect size = –1.05; 95% CI [–1.94 to –0.149]). These data are consistent with our findings of increased insulin sensitivity in the face of decreased CYP8B1 activity in the *CYP8B1* mutation carriers.

### Lower insulin and GLP-1 response during MMTT in CYP8B1 mutation carriers.

We next assessed whether increased β cell function contributed to the increased whole-body insulin sensitization in the mutation carriers. The disposition index, a measure of β cell function (10) during MMTT, was unchanged (*P* = 0.65) (Figure 4E). Glucagon-like peptide 1 (GLP-1), an incretin hormone, potentiates β cell insulin secretion in response to glucose stimulation (11). Both fasting GLP-1 (controls, 34.3 [26.2]; carriers, 22.9 [18.9] pmol/L; *P* = 0.04) and GLP-1 during MMTT (AUC: controls, 5847.0 [3051.5]; carriers, 3572.0 [2201.0]; *P* = 0.04) were decreased (Figure 4, F and G), in line with the reduced insulin levels observed during MMTT. These data show that the lower insulin secretion in mutation carriers maybe in response to improved peripheral insulin sensitivity, to maintain normoglycemia.

### BA composition from CYP8B1 mutation carriers increases muscle insulin signaling.

**Figure 5:**
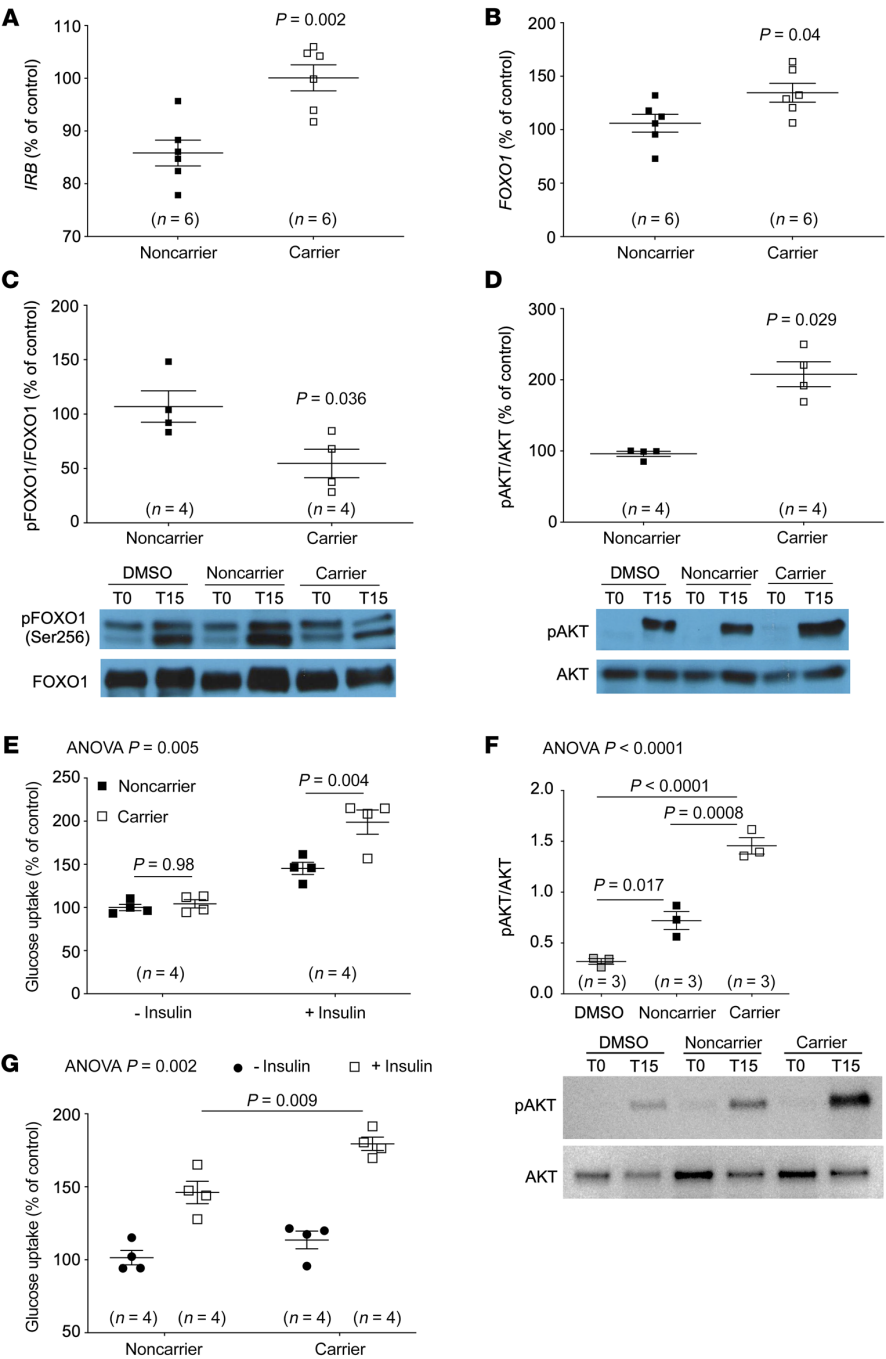
Bile acids act directly on the skeletal muscle to increase insulin signaling. Primary human skeletal muscle cells exposed to the CA/CDCA ratio from subjects with highest and lowest insulin sensitivity showed (**A**) increased expression of the muscle insulin receptor isoform B (*IRB*), (**B**) increased expression of the bile acid–associated transcription factor forkhead box O1 (*FOXO1*), a transcription factor regulating insulin receptor levels, (**C**) decreased FOXO1 phosphorylation, indicating increased FOXO1 transcription activity, (**D**) increased AKT phosphorylation, indicating increased insulin signaling, and (**E**) increased 2-deoxyglucose uptake in muscle cells exposed to carrier CA/CDCA ratio. Increased (**F**) AKT phosphorylation and (**G**) 2-deoxyglucose uptake in skeletal muscle cells exposed to the median CA/CDCA ratio from mutation carriers compared to those exposed to the median CA/CDCA ratio of controls. Data are shown as mean ± SEM. CA, cholic acid; CDCA, chenodeoxycholic acid. Data in **A**–**D** and **F** were quantified 15 minutes after insulin stimulation, and data in **E** and **G** were quantified 1 hour after insulin stimulation. Data were assessed using unpaired *t* tests (**A**–**C**, normally distributed data), the Mann-Whitney *U* test (**D**), 1-way ANOVA followed by Tukey’s multiple comparison test (**F**), or 2-way ANOVA with Tukey’s post hoc test (**E** and **G**). In **E**–**G**, the overall ANOVA *P* value is shown on the top left of the graphs, and individual post hoc test *P* values are shown for the relevant pairwise comparisons. See complete unedited blots in the supplemental material.

Because our data indicated that reduced CYP8B1 activity mainly affects peripheral insulin sensitivity in humans, and skeletal muscle is the principal site of insulin-mediated glucose clearance (12), we determined whether increased muscle insulin sensitization contributed to the observed insulin sensitivity. Human skeletal muscle cells were incubated with CA and CDCA at the ratios of the individuals with the highest (CA/CDCA = 2:23) and lowest insulin sensitivities (CA/CDCA = 21:29). The human insulin receptor has 2 isoforms, A and B, with *IRB* being the predominant isoform in insulin target tissues, including muscle (13). *IRB* expression was increased with the carrier BA ratio (noncarrier, 85.8% ± 2.5%; carrier, 100.1% ± 2.5% vs. control; *P* = 0.002) (Figure 5A), suggesting that the BA composition in mutation carriers may directly impact skeletal muscle insulin signaling. Insulin receptor expression is regulated by the BA-associated transcription factor forkhead box O1 (FOXO1) (14). *FOXO1* expression was upregulated in muscle cells exposed to the carrier BA ratio (noncarrier, 106.1% ± 8.3%; carrier, 134.6% ± 8.8% vs. control; *P* = 0.04) (Figure 5B). Phosphorylated FOXO1(S^256^) is cytoplasmic, whereas dephosphorylated FOXO1 is retained in the nucleus where it functions as a transcription factor (14). Phospho(S^256^)FOXO1 was decreased in muscle cells exposed to the carrier BA mix in response to insulin, suggesting increased nuclear localization and transcriptional activity of FOXO1 in carriers (noncarrier, 107.0% ± 14.4%; carrier, 54.7% ± 13.1% vs. control; *P* = 0.036) (Figure 5C). Phospho-AKT is an established marker of insulin signaling (15). Phospho(S^473^)AKT was increased by 116% in muscle cells treated with the carrier CA/CDCA ratio in response to insulin (noncarrier, 96.1% ± 3.6%; carrier, 207.9% ± 17.6% vs. control; *P* = 0.029) (Figure 5D). Additionally, 2-deoxyglucose uptake was increased in muscle cells exposed to the carrier BA ratio in response to insulin (noncarrier + insulin, 145.3% ± 7.1%; carrier + insulin, 198.9% ± 14.1% vs. control; *P* = 0.004) (Figure 5E), confirming that the BA composition in *CYP8B1* mutation carriers led to significantly increased muscle cell insulin signaling and glucose uptake.

These experiments utilized CA and CDCA ratios of individuals with the highest (CA/CDCA = 2:23) and lowest insulin sensitivities (CA/CDCA = 21:29). Thus, we assessed whether the median CA/CDCA ratio of the mutation carriers (CA/CDCA = 4.5:45.5) would increase muscle cell insulin signaling and glucose uptake when compared with the median CA/CDCA ratio of noncarrier controls (CA/CDCA = 8.3:41.7). Phospho(S^473^)AKT was increased approximately 2-fold in muscle cells treated with the median carrier CA/CDCA ratio in response to insulin (p-AKT/AKT: noncarrier, 0.72 ± 0.09; carrier, 1.46 ± 0.08; *P* = 0.0008) (Figure 5F). In line with this, the uptake of 2-deoxyglucose was increased in muscle cells treated with the median carrier CA/CDCA ratio in response to insulin (noncarrier +insulin, 146.1% ± 7.7%; carrier + insulin, 179.5% ± 4.5% glucose uptake vs. control; *P* = 0.009) (Figure 5G). These data further confirm that the CA/CDCA ratio in carriers increases insulin signaling in human skeletal muscle cells compared with noncarrier controls.

### CDCA increases glucose uptake in skeletal muscle cells.

**Figure 6:**
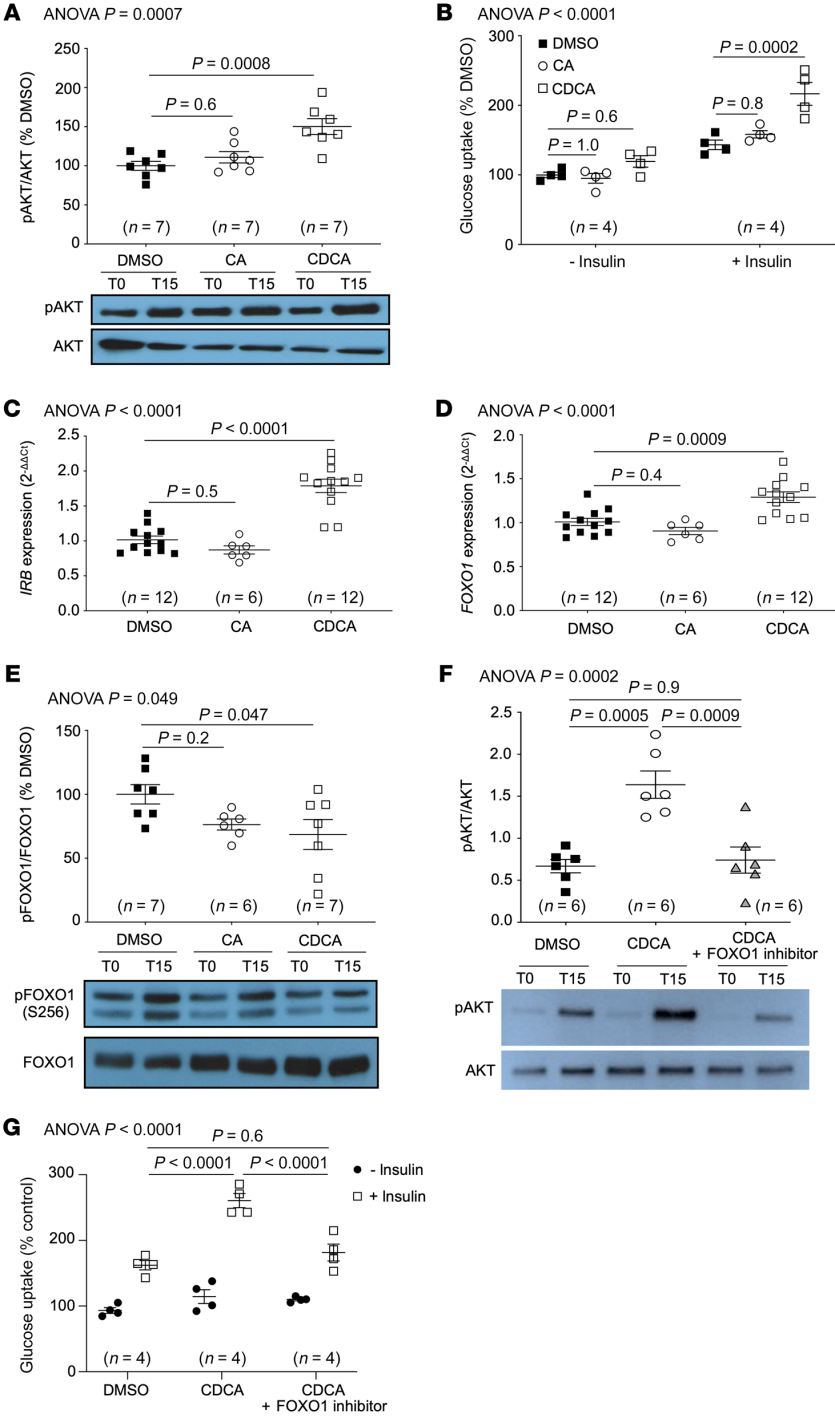
CDCA increases insulin signaling and glucose uptake in skeletal muscle cells. Exposure of skeletal muscle cells to CDCA increases (**A**) phospho-AKT, indicating increased insulin signaling, and (**B**) 2-deoxyglucose uptake. CDCA also increases muscle cell (**C**) insulin receptor (*IRB*) expression, (**D**) expression of its transcription factor, forkhead box O1 (*FOXO1*), and (**E**) FOXO1 activity, indicated by decreased FOXO1 phosphorylation. These effects on muscle cell insulin sensitivity and glucose uptake are modulated via FOXO1, since inhibition of FOXO1 with the FOXO1-specific inhibitor AS1842856 attenuated the CDCA-mediated increase in (**F**) insulin signaling, determined by quantification of phospho-AKT, and (**G**) muscle cell glucose uptake in response to insulin. CA, cholic acid; CDCA, chenodeoxycholic acid. Data in **A** and **C**–**F** were quantified 15 minutes after insulin stimulation. Data in **B** and **G** were quantified 1 hour after insulin stimulation. Data were analyzed using 1-way ANOVA followed by Tukey’s multiple comparison test (**A** and **C**–**F**) or 2-way ANOVA followed by Tukey’s test (**B** and **G**). In all panels, the overall ANOVA *P* value is shown on the top left of the graphs, and individual post hoc test *P* values are shown for the relevant pairwise comparisons. See complete unedited blots in the supplemental material.

The ratio of CDCA and its conjugates to total BAs was increased in heterozygous human *CYP8B1* mutation carriers, suggesting that increased CDCA may be sufficient to increase muscle insulin sensitivity. Thus, we next assessed whether CDCA alone was sufficient to increase muscle insulin signaling. Exposure of muscle cells to CDCA, but not CA, increased phospho(S^473^)AKT in response to insulin (control, 100.0% ± 5.7%; CDCA, 150.2% ± 10.2%; *P* = 0.0008; CA, 111.0% ± 7.3% vs. control; *P* = 0.6) (Figure 6A), indicating increased insulin signaling. Muscle cell uptake of 2-deoxyglucose was also increased in response to CDCA, but not CA (control + insulin, 143.3% ± 6.9%; CDCA + insulin, 216.5% ± 16.4%; *P* = 0.0002; CA + insulin, 158.3% ± 5.2%; *P* = 0.8) (Figure 6B). Moreover, exposure of myotubes to CDCA resulted in increased *Insr* and *Foxo1* expression (Figure 6, C and D), and decreased phospho(S^256^)FOXO1 levels (control, 100.0% ± 7.6%; CDCA, 68.6% ± 11.8%; *P* = 0.047; CA, 76.4% ± 4.3%; *P* = 0.2) (Figure 6E). To further confirm a direct effect of CDCA on muscle insulin signaling, we performed dose-response experiments. Increasing doses of CDCA significantly increased insulin signaling, assessed by phospho(S^473^)AKT levels (ANOVA *P* < 0.0001; Supplemental Figure 1A), and increased muscle cell 2-deoxyglucose uptake (ANOVA *P* < 0.0001; Supplemental Figure 1B), in response to insulin stimulation.

In addition to CA, levels of circulatory DCA were largely decreased in the *CYP8B1* mutation carriers (Supplemental Table 2). However, as with CA, exposure of muscle cells to 10 μmol/L DCA, the highest concentration not toxic to muscle cells, did not result in altered levels of phospho(S^473^)AKT or glucose uptake (Supplemental Figure 1, C and D) in response to insulin.

### Inhibition of FOXO1 reverses the beneficial effects of CDCA on muscle insulin signaling.

Our data suggest that a CDCA-mediated increase in muscle FOXO1 activity may contribute to its role in increasing insulin sensitivity. Thus, we assessed the impact of FOXO1 inhibition on CDCA’s ability to increase muscle insulin sensitivity. Treatment of muscle cells with the FOXO1-specific inhibitor AS1842856 (16) attenuated the effects of CDCA on both muscle insulin signaling, assessed by quantifying the phospho(S^473^)AKT/AKT ratio (control, 0.67 ± 0.08; CDCA, 1.64 ± 0.16; *P* = 0.0005; CDCA + FOXO1 inhibitor, 0.74 ± 0.16; *P* = 0.9) (Figure 6F), and on muscle 2-deoxyglucose uptake in response to insulin (control + insulin, 162.2% ± 7.1%; CDCA + insulin, 260.6% ± 10.7%; *P* < 0.0001; CDCA + FOXO1 inhibitor + insulin, 181.5% ± 13.0% vs. control; *P* = 0.6) (Figure 6G). These data suggest that CDCA directly increases insulin signaling and glucose uptake in skeletal muscle cells, and also suggest that modulation of FOXO1 activity may represent a pathway through which CDCA increases muscle insulin sensitivity.

## Discussion

We show here that haploinsufficiency of *CYP8B1* improves insulin sensitivity in humans. Although BA synthesis involves several enzymes, and complex feedback and feedforward mechanisms are involved in the regulation of BA metabolism (1), the reduction in CYP8B1 function is not compensated for, resulting in increased insulin sensitivity in heterozygous *CYP8B1* mutation carriers.

Our data suggest that a CDCA-mediated increase in skeletal muscle insulin signaling contributed to the phenotype in the *CYP8B1* mutation carriers. BAs have been shown to modulate insulin sensitivity via increasing β cell insulin secretion or altering hepatic glucose metabolism (17, 18). We excluded these processes as contributing to the increased insulin sensitivity in the *CYP8B1* mutation carriers. Our findings suggest a change in concept, where the BA CDCA modulates insulin sensitivity by increasing skeletal muscle insulin signaling.

CDCA is a highly potent endogenous agonist of FXR (19), a modulator of glucose metabolism. Thus, a mechanism by which reduced CYP8B1 activity may increase muscle insulin sensitivity is through increasing FXR signaling. However, FXR is not found in muscle (18), excluding a direct role for FXR in muscle insulin sensitization. Both hepatic and intestinal FXR modulate glucose metabolism through regulating hepatic insulin sensitivity and glucose production, in part via the FGF19 pathway. However, hepatic insulin sensitivity and glucose production were unchanged in the human mutation carriers. Reducing FXR activity was shown to decrease skeletal muscle insulin sensitivity in mice through an as yet unclear mechanism (18), suggesting that FXR cannot be excluded as indirectly contributing to the improved muscle insulin sensitization.

As opposed to FXR, TGR5, which can also be activated by CDCA, is expressed in skeletal muscle. Overexpression of TGR5 in skeletal muscle cells increased energy expenditure (20), suggesting a mechanism by which muscle TGR5 might modulate glucose metabolism. However, the human *CYP8B1* mutation carriers showed unchanged energy expenditure (Supplemental Figure 2), as did *Cyp8b1^–/–^* mice in a previous study (2). In line with this, muscle-specific overexpression of TGR5 did not increase insulin sensitivity in mice (21). The same study found that, in primary mouse myotubes, stimulation of TGR5 with its most potent BA agonist LCA did not increase the insulin signaling pathway. Together, these data suggest that CDCA is unlikely to increase muscle insulin signaling via the activation of TGR5. Independent of the muscle, BA-activated TGR5 also improves glucose metabolism by increasing GLP-1 levels and β cell insulin secretion (22). However, we found decreases in both GLP-1 levels and insulin secretion in our *CYP8B1* mutation carriers, suggesting that the known TGR5-mediated mechanisms are unlikely to contribute to the improved insulin sensitivity we observe.

How extracellular CDCA modulates intracellular signaling in the muscle is unclear. Known BA transporters are not highly expressed in skeletal muscle (http://biogps.org/). It is possible that CDCA modulates intracellular signaling through as yet unidentified muscle-specific cell surface receptors or BA transporters. Our data suggest that CDCA modulates muscle insulin signaling through increasing muscle FOXO1 activity, and inhibition of FOXO1 reversed the increased CDCA-mediated muscle insulin signaling. How CDCA decreases muscle FOXO1 phosphorylation, thus increasing its nuclear retention and transcriptional activity, remains unclear. One possible mechanism by which CDCA may reduce phospho-FOXO1 is by increasing the activity of phosphatases acting on FOXO1. BAs do modulate other phosphatases such as Src-homology 2 domain–containing tyrosine phosphatase 2 (SHP2) (23).

The reason for the potential selectivity of CDCA for skeletal muscle in regulating insulin sensitivity is unclear. However, FOXOs show tissue-specific protein interactions to modulate their functions in metabolic regulation (24). Distinct interacting proteins modulating FOXO1’s nuclear entry and therefore its transcriptional activity have been described in adipocytes, liver, pancreas, skeletal muscle, cardiac muscle, and hypothalamus (24). Further studies are needed to determine whether CDCA modulates the muscle-specific regulators of FOXO1 function.

Considering preclinical findings supporting FXR agonists in regulating glucose and lipid metabolism, obeticholic acid (OCA), an analog of CDCA, was assessed in type 2 diabetics (4). Increased GIR, the primary endpoint, was met (4). However, OCA increased LDL-C and decreased HDL-C (25), suggesting an adverse induction of a proatherogenic lipid profile. *CYP8B1* mutation carriers showed decreased total cholesterol/HDL-C and APOB/APOA-I ratios, suggesting that CYP8B1 inhibition may reduce type 2 diabetes without proatherogenic lipid changes. In addition, hs-CRP levels were decreased by approximately 50%, and hepatic fat was decreased by 30% in the *CYP8B1* mutation carriers. These observations are consistent with those in *Cyp8b1^–/–^* mice, which showed increased HDL-C, decreased LDL-C, and reduced atherosclerotic lesions when fed atherogenic diets (26, 27). Additionally, *Cyp8b1* depletion prevented the progression of hepatic steatosis and caused its regression (28), suggesting that inhibiting CYP8B1 may reduce susceptibility to atherosclerosis as well as hepatic steatosis.

The CYP8B1 product, 7α,12α-dihydroxy-4-cholesten-3-one, was not decreased in plasma of mutation carriers. However, downstream 12α-BAs were decreased by 52%, suggesting that the 12α-hydroxylase function of CYP8B1 in the liver was indeed reduced. Significantly increased unconjugated CDCA was also not observed in mutation carriers, although the ratio of CDCA and its conjugates to total BAs was increased. The reasons for this are unclear. In the mutation carriers, plasma BAs were quantified after an overnight fast. However, it has been shown that circulatory BA levels increase postprandially, with CDCA showing the largest increase (up to 5-fold) (29). Furthermore, CDCA can be converted to CA (30), suggesting that this pathway may have been upregulated under low CA conditions in heterozygous *CYP8B1* mutation carriers.

*Cyp8b1^–/–^* mice are viable, with no apparent adverse phenotypes, suggesting that the absence of CYP8B1 may not be harmful. We did not identify compound heterozygous or homozygous carriers of CLOF mutations, and almost all CLOF mutations were identified in only 1 or 2 heterozygotes. Thus, homozygous or compound heterozygous CLOF mutation carriers are likely to be extremely rare. One CLOF mutation, *R26X*, was found at 1.6% frequency in Malays. Assuming Hardy-Weinberg equilibrium, sequencing of approximately 13,000 Malays is required to identify a single *R26X* homozygote. Thus, it is unsurprising that we identified no homozygous or compound heterozygous CLOF mutation carriers. Additionally, in a large publicly aggregated database (gnomAD), no individuals harboring homozygous predicted CLOF mutations were reported, confirming that these individuals are extremely rare.

We establish a fundamental role for CYP8B1-mediated changes in BA composition in the regulation of peripheral insulin sensitivity. We show that reduced activity of CYP8B1 is efficacious in increasing insulin sensitivity in humans, and mechanistically link CDCA signaling to muscle insulin sensitivity. We demonstrate here a target for future therapeutic intervention for diabetes.

## Methods

Detailed methods are provided in the supplementary material.

### Identification of carriers of CLOF CYP8B1 mutations.

The *CYP8B1* coding region was Sanger sequenced in population cohorts of nondiabetics from the Singapore Eye Research Institute and the Saw Swee Hock School of Public Health, National University of Singapore. Sequences were assembled in Sequencher (Gene Codes) and aligned to the human *CYP8B1* reference sequence (GenBank AF090320.1). All nonsynonymous variants, frameshifts, and insertions/deletions were generated in human *CYP8B1* cDNA and functionally characterized.

### In vitro functional testing of CYP8B1 variants.

*CYP8B1* cDNA inserted into pcDNA3.1^(+)^ (Invitrogen) was used as the template for site-directed mutagenesis. The neomycin resistance cassette in pcDNA3.1^(+)^ was replaced with green fluorescent protein (*Gfp*) in order to assess transfection efficiency of the variants. Mutagenesis primers were designed for each of the *CYP8B1* variants, and PCR performed with conditions adjusted for each primer pair, amplifying the entire plasmid. PCR products were isolated from the template by Dpn1 digestion, transformed into DH5α competent cells, and amplified plasmids were extracted by E.Z.N.A. Plasmid Mini Kit II (Omega Bio-tek Inc). The mutant *CYP8B1* cDNAs were sequence confirmed, subcloned into the pcDNA3.1^(+)^-*Gfp* vector, and used for the in vitro assay. For the in vitro functional assay, HEK293T (ATCC) cells in 6-well plates were transiently transfected with the plasmids harboring wild-type *CYP8B1*, variant *CYP8B1*, or empty vector using X-tremeGENE HP (Roche). After 24 hours, fresh DMEM with 10% FBS containing 10 μmol/L of the CYP8B1 substrate 7α-hydroxy-4-cholesten-3-one (Toronto Research Chemicals) was added to the cells at 3 mL/well. After 4 hours, media were collected, centrifuged, and frozen until quantification of 7α,12α-dihydroxy-4-cholesten-3-one by LC/MS (Experimental Therapeutics Centre, Singapore). Cells were washed, centrifuged, and frozen for Western immunoblotting.

### Recruitment of study cohort.

Carriers of *CYP8B1* mutations and age-, sex-, race-, and BMI-matched nonmutation carrier controls from the same cohorts were recruited at a ratio of 1 carrier to 2 controls for metabolic studies. Five participants only had 1 matched control. The studies of BAs and insulin phenotypes excluded individuals with BMI greater than 30, the World Health Organization definition of obesity, since obesity modulates insulin sensitivity. Individuals were also excluded if they had type 2 diabetes mellitus, renal impairment, elevated serum aspartate aminotransferase or alanine aminotransferase, chronic liver disease, or medications or previous gastrointestinal surgery that may alter glucose or BA metabolism (Supplemental Figure 3).

### Plasma BA quantification.

BAs and hydroxyl cholestenones (7α-hydroxy-4-cholesten-3-one and 7α,12α-dihydroxy-4-cholesten-3-one) were measured in plasma following overnight fasts using ultraperformance liquid chromatography–multiple reaction monitoring/mass spectroscopy (UPLC-MRM/MS) (University of Victoria-Genome BC Proteomics Centre) as described previously (31).

### MMTT.

After 8-hour fasts, venous blood was collected for plasma glucose, insulin, and GLP-1 measurements at 0, 15, 30, 60, 90, and 120 minutes after ingestion of liquid mixed meal (Ensure Plus: 55% carbohydrate, 30% fat, 15% protein at 6 kcal/kg, max 360 kcal).

### Hyperinsulinemic-euglycemic clamps.

Participants ingested 2 doses of ^2^H_2_O (total dose of 3 g/kg) for quantification of gluconeogenesis and glycogenolysis. To measure hepatic glucose production, primed-constant infusion of [6,6-^2^H_2_]-glucose was performed. Insulin was infused at 40 mU/m^2^ body surface area/min for 180 minutes. Blood glucose was measured every 5 minutes. Blood for plasma insulin measurement was obtained every 30 minutes. Dextrose 20% (wt/vol) enriched with [6,6-^2^H_2_]-glucose was infused at a variable rate to maintain blood glucose at 100 mg/dL with a coefficient of variation of less than 5%.

### Associations of CYP8B1 mutations with diabetes phenotypes in the T2D Knowledge Portal.

Association analyses for the CLOF *CYP8B1* mutant *R26X*, selected because it was the most frequent in our study cohort, were performed using the publicly available Type 2 Diabetes Knowledge Portal (http://www.type2diabetesgenetics.org). Associations between *R26X* and fasting insulin, adjusted for BMI, age, and sex were calculated using the portal’s Genetic Association Interactive Tool (GAIT), in which single-variant and gene-level association analysis can be conducted in 45,231 exomes from the AMP-T2D-GENES study.

### Skeletal muscle cell experiments.

Human adult skeletal muscle cells (Cell Applications) were differentiated following manufacturer’s instructions and treated with a 50 μmol/L CA/CDCA mixture at the ratio of carrier or control, or dimethyl sulfoxide (DMSO) for 24 hours. To quantify AKT and FOXO1 phosphorylation subsequent to insulin stimulation, differentiated cells were treated with 100 nmol/L insulin (Humalog, Eli Lilly). For glucose uptake assays, BA-treated cells were incubated with and without 100 nmol/L insulin, glucose uptake was quantified using fluorescence, and protein content was determined for normalization of glucose uptake.

### Statistics.

All human data were first assessed for normality. Non-normal data were log transformed. Normal data are reported as mean ± SEM and were analyzed using parametric unpaired *t* tests. Non-normal data are reported as median (IQR) and were analyzed using the nonparametric Mann Whitney *U* test. Fisher’s exact tests were used for categorical data, 1-way ANOVA was used for analyses of more than 2 groups, and 2-way ANOVA was used for repeated measures. All tests were 2-sided, and performed using GraphPad Prism 9.0. A *P* value of less than 0.05 was considered significant.

### Study approval.

The human study received approval from the SingHealth Centralized Institutional Review Board. All participants provided written informed consent prior to the study.

## Author contributions

RRS, MRH, AKG, and HCT conceptualized the study. RRS and HCT acquired funding. RRS, HCT, FK, JLG, RMVD, RST, KAR, and CYC supervised the study. RRS wrote the original draft of the manuscript. SZ, RC, MC, DCM, BJC, KPS, THVD, JP, LJT, SVH, BR, PC, JLG, SC, JF, and XS conducted the investigation and analyses. JLG, AKG, FK, SC, JF, KAR, XS, HCT, and RRS provided resources. SZ, AKG, FK, CD, HCT, and MRH reviewed and edited the manuscript.

## Supplementary Material

Supplemental data

ICMJE disclosure forms

## Figures and Tables

**Table 1:**
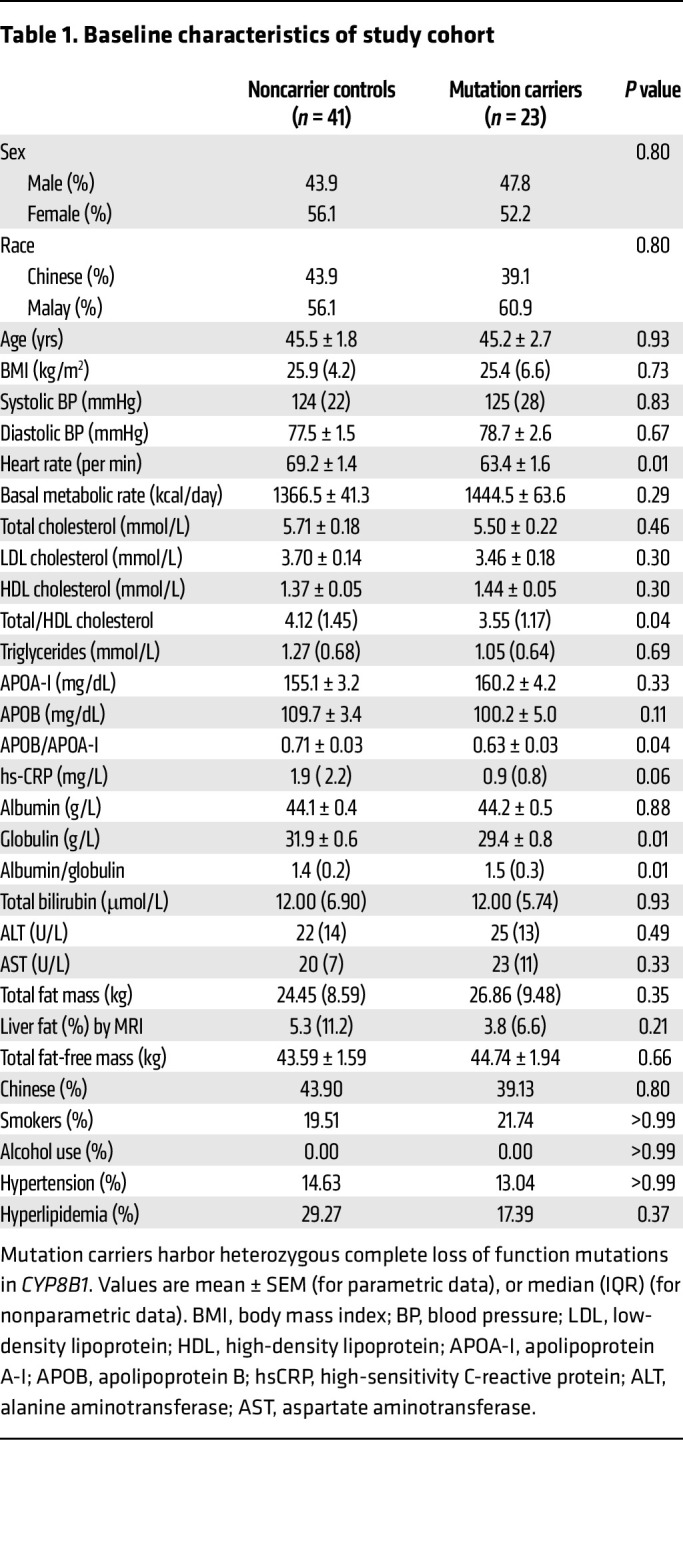
Baseline characteristics of study cohort

## Version 1 09/15/2022

In-Press Preview

## Version 2 11/01/2022

Electronic publication
