# Supplementary material for: Haploinsufficiency of CYP8B1 associates with increased insulin sensitivity in humans
Source: J Clin Invest. 2022 Nov 1;132(21):e152961. doi: 10.1172/JCI152961 (PMC9621133; doi:10.1172/JCI152961)

SUPPLEMENTARY ONLINE DATA

Haploinsufficiency for *CYP8B1* associates with increased insulin sensitivity in humans

Shiqi Zhong[1,2], Raphael Chevre[2*], David Castano Mayan[1,2,3], Maria Corlianò[1,2,3], Blake Cochran[4], Kai Ping Sem[2], Theo H. van Dijk[5], Jianhe Peng[6,§], Liang Juin Tan[2], Siddesh V. Hartimath[7], Boominathan Ramasamy[7], Peter Cheng[7], Albert K. Groen[5], Folkert Kuipers[5], Julian L. Goggi[7], Chester Drum[1,3], Rob M. van Dam[8], Ru San Tan[9], Kerry-Anne Rye[4], Michael R. Hayden[10], Ching-Yu Cheng[11,12], Shaji Chacko[13], Jason Flannick[14-17], Xueling Sim[8], Hong Chang Tan[18,#], Roshni R. Singaraja[1,2,3,#]

[1]Department of Medicine, Yong Loo Lin School of Medicine, National University of Singapore, Singapore.
[2]Translational Laboratory in Genetic Medicine, Agency for Science, Technology and Research (A*STAR), Singapore.
[3]Cardiovascular Research Institute, National University Health System, Singapore.
[4]School of Medical Sciences, University of New South Wales, Sydney, Australia.
[5]Departments of Pediatrics and Laboratory Medicine, University of Groningen, University Medical Center Groningen, The Netherlands.
[6]Experimental Therapeutics Centre, A*STAR, Singapore.
[7]Singapore Bioimaging Consortium, A*STAR, Singapore.
[8]Saw Swee Hock School of Public Health, National University of Singapore and National University Health System, Singapore.
[9]Department of Cardiology, National Heart Centre, Singapore.
[10]Department of Medical Genetics, Centre for Molecular Medicine and Therapeutics, The University of British Columbia, Vancouver, British Columbia, Canada.
[11]Singapore Eye Research Institute, Singapore National Eye Centre, Singapore.
[12]Ophthalmology and Visual Sciences Academic Clinical Program (Eye ACP), Duke-NUS Medical School, Singapore.
[13]USDA/ARS Children's Nutrition Research Centre, Department of Pediatrics, Baylor College of Medicine, Houston, Texas, USA.
[14]Program in Metabolism, Broad Institute, Cambridge, MA, USA.
[15]Program in Medical & Population Genetics, Broad Institute, Cambridge, MA, USA.
[16]Division of Genetics and Genomics, Boston Children's Hospital, Boston, MA, USA
[17]Department of Pediatrics, Harvard Medical School, Boston, MA, USA
[18]Department of Endocrinology, Singapore General Hospital, Singapore.

[*]Present address: Institute of Experimental Pathology, Center for Molecular Biology of Inflammation, Westfälische Wilhelms-Universität, Münster, Germany.

[§]Present address: Culture Collections, Public Health England, London, UK
[#]These authors contributed equally

Content ***Page number***

Methods 3

Sequencing/*In vitro* testing of *CYP8B1* variants 3

Subject recruitment and baseline screening 4

Stable-Isotope Tracers 7

Hyperinsulinemic-euglycemic clamp 7

Measurement of isotopic enrichment 8

Calculation of total glucose rate of appearance and glucose production 8

Measurement of gluconeogenesis and glycogenolysis 9

Measurement of insulin sensitivity 9

T2D Knowledge Portal associations 10

In vitro skeletal muscle cell culture and transcript quantification 10

Skeletal muscle cell phospho-AKT and phospho-FOXO1 quantification 12

Skeletal muscle cell glucose uptake 13

Supplemental Figure 1 14
Dose response to CDCA and response to deoxycholic acid in skeletal muscle cells

Supplemental Figure 2 16
Baseline resting energy expenditure (REE) is not altered in heterozygous *CYP8B1* mutation carriers

Supplemental Figure 3 17
Study flowchart

Supplemental Table 1 18
Predicted function and assay-verified *in vitro* function of all *CYP8B1* variants

Supplemental Table 2 28
Plasma bile acid profile of heterozygous *CYP8B1* mutation carriers and controls

References 31

Methods:

Sequencing of *CYP8B1* in cohorts

Genomic DNA samples were obtained from population cohorts from the Singapore Eye Research Institute and the School of Public Health, National University of Singapore (SEED: Singapore Epidemiology of Eye Disease cohort (1), MEC: Singapore Multi-Ethnic Cohort (2), SH2012: Singapore Health 2012 cohort (3)). *CYP8B1* was amplified by PCR (fwd: 5´ACTTTCCAAGGCCTCCCAAAG, rev: 5´GGTGAGAACAGGTGAGAGATGC), and Sanger sequenced (GATC Biotech AG, Germany) using four sets of primers: set 1: fwd: 5´ACTTTCCAAGGCCTCCCAAAG, rev: 5´GTGAACACATCCCCATGCTT; set 2: fwd: 5´GGTGGTCATTGCTGGATACC, rev: 5´GAAGGTCAAACTTGCGGAAC; set 3: fwd: 5´ACACGAAGGACAAGGAGCAG, rev: 5´GTGGATGTCAGGGTCCATGT; set 4: fwd 5´CCCTCCTCAGGTTGGTTCAT, rev: 5´GGTGAGAACAGGTGAGAGATGC. Sequences were assembled in Sequencher (Gene Codes Corporation, USA) and aligned to the human *CYP8B1* reference sequence (GenBank: AF090320.1). All polymorphisms, frameshift and insertion/deletion variants were recorded.

In vitro functional testing of *CYP8B1* variants

Generation of mutants is described in the main manuscript. For LC/MS analysis of 7α,12α-dihydroxy-4-cholesten-3-one in the media, 100 μl of media was transferred to an eppendorf tube with 300 μl of internal standard (1 μmol/L of 7α-hydroxy-4-cholesten-3-one-d7 in acetonitrile, Toronto Research Chemicals, cat# H825133), vortexed, centrifuged, and 300 μl of supernatant was transferred to 96-deep well plates, dried and resuspended in 100 μl of 50% methanol/40% H_2O+10%

TFA (trifluoroacetic acid) for LC/MS. Mobile phases A = 0.1% formic acid in water, and B = 0.1% formic acid in acetonitrile were used with an injection volume of 10 μl. Peak area ratio was calculated by the division of peak area (PA) for 7α,12α-dihydroxy-4-cholesten-3-one by PA of the internal standard 7α-hydroxy-4-cholesten-3-one-d7.

For western blotting, cells were lysed in extraction buffer (25 mmol/L NH_4Cl, pH 8.2; 5 mmol/L EDTA; 0.4 g/L SDS; 8 g/L Triton X-100; 1x protease inhibitor from Roche), centrifuged, and 20 μg of proteins were resolved on 10% polyacrylamide gels, transferred to PVDF membranes, blocked with 5% skim milk, and probed with anti-CYP8B1 (C-term, Abgent # AP8787B) or anti-GAPDH (Sigma-Aldrich #SAB2108668) antibodies, followed by horseradish peroxidase–conjugated secondary antibodies (Santa Cruz Biotechnology). Blots were developed by chemiluminescence (Bio-Rad). Relative densities of CYP8B1 to calnexin were quantified by ImageJ.

Subject recruitment and baseline screening

Carriers of complete loss of function *CYP8B1* mutations and age-, gender-, race- and BMI-matched non-mutation carrier controls were recruited from the same population cohorts with a ratio of 1 mutation carrier to 2 controls for metabolic studies at the SingHealth Investigational Medical Unit. This study received approval from the SingHealth Centralized Institutional Review Board and all subjects provided written informed consent before enrolment into the study. Subject inclusion and exclusion criteria are listed below. Subjects underwent mixed-meal tolerance testing (MMTT) and hyperinsulinemic-euglycemic clamps with stable-isotope glucose infusion for quantification insulin sensitivity. Body composition was measured using

dual X-Ray absorptiometry (Hologic Discovery Wi densitometer, Hologic Inc, Massachusetts, USA), and liver fat was measured using MRI.

Subjects were included or excluded based on the following criteria

Inclusion Criteria

Subjects must have met all the inclusion criteria listed below to participate in the study

1. Age: 21-65 years
2. Able to provide informed consent
3. Known *CYP8B1* mutation status

Exclusion criteria

All subjects meeting any of the exclusion criteria listed below at baseline were excluded from participation.

1. Diabetes Mellitus (diagnosed according to 2014 Singapore Ministry Of Health Clinical Practice Guidelines for Diabetes Mellitus)
2. Renal impairment (glomerular filtration rate < 60 ml/min)
3. Serum alanine aminotransferase or aspartate aminotransferase > 3x upper limit of normal of laboratory reference range
4. Clinically significant anemia (Hb < 10 g/dL)
5. Known allergy to insulin
6. Pregnancy
7. Surgery requiring general anaesthesia within 6-weeks before enrolment
8. Significant cardiovascular disease (acute myocardial infarction, congestive cardiac failure, ischemic heart disease)
9. Dysrhythmia (atrial fibrillation, sick sinus syndrome, supraventricular tachycardia)

10. Previous stroke
11. Uncontrolled hypertension (BP > 180/110 mmHg)
12. Peripheral vascular disease (large vessel)
13. Chronic hepatitis
14. Inflammatory bowel disease (Crohn's or UC)
15. Dementia
16. Active psychosis
17. Depression requiring medication
18. Suicide attempt(s)
19. Daily consumption of alcohol or liver cirrhosis
20. Drug abuse in past 6-months
21. Present systemic steroid usage (eg. prednisolone, hydrocortisone, cortisone, dexamethasone)
22. Malignancy within 5-years (except squamous cell and basal cell cancer of the skin)
23. Uncontrolled thyroid disease
24. Cholestatic Liver Diseases (eg. Primary Biliary Cirrhosis, Primary Sclerosing Cholangitis)
25. Gastrointestinal surgery that alters bile acid metabolism including bariatric surgery, pancreatectomy, colectomy, small intestine surgery, cholecystectomy within 5 years.
26. Present intake of medications that interfere directly with bile acid metabolism
27. Present intake of medications that interfere directly with glucose metabolism
28. Prescription weight loss medications

Stable-Isotope Tracers

Sterile and pyrogen-free deuterium oxide (^2H_2O), 99% enriched and [6,6-$^2H_2$] glucose, 99% enriched were purchased from Cambridge Isotope Laboratories (Andover, MA, USA). [6,6-$^2H_2$] glucose was dissolved in isotonic saline, and the solutions was filtered through a 0.2 μm filter into sterile syringes prior to administration. ^2H_2O was administered orally without additional preparation.

Hyperinsulinemic-euglycemic clamp

All subjects were admitted to the SingHealth Investigational Medical Unit the evening before hyperinsulinemic-euglycemic clamps. Subjects were fed a standardized dinner consisting of 1/3 of their total daily calorie requirement with macronutrient composition reflective of the typical Singaporean dietary intake (30% fat, 55% carbohydrate and 15% protein) and fasted from 10 pm (except plain water) until the completion of clamp studies. Intravenous catheters were inserted on opposite arms for blood sampling and intravenous infusion.

Study subjects consumed two doses of ^2H_2O (total dose of 3g/kg) at 10 pm and 12 am on the night of admission for quantification of gluconeogenesis and glycogenolysis. To measure hepatic glucose production (HGP), primed-constant rate of [6,6-$^2H_2$] glucose (2 mg/kg, 3 mg/kg/min) was infused from 7.30 am until the completion of study at 12.30 pm. Insulin was infused at 40 mU/m^2 (240 pmol/m^2) body surface area/min for 180 minutes from 9.30 am until 12.30 pm. Blood glucose concentrations were measured every 5 minutes using an on-site glucose analyzer (YSI 2300 STATPLUS, YSI Incorporated, Life Sciences, USA). Blood for plasma insulin

measurement was obtained at every 30 minutes. Dextrose 20%, enriched with [6,6-$^2H_2$] glucose was infused at a variable rate to maintain blood glucose at 100 mg/dL (5.6 mmol/L) with a coefficient of variation of <5%.

Measurement of isotopic enrichment

The isotopic enrichment of glucose during fasting and final 30 minutes of the clamp was measured at the Stable Isotope Core Laboratory of the Children's Nutrition Research Center, Baylor College of Medicine, Houston, Texas. To avoid analytical variation, serum and plasma were stored at -80°C and analyzed as a single batch after completion of the study. Penta-acetate derivative of glucose was prepared, and the isotopic enrichment of [6,6-$^2H_2$] glucose was measured by GC-MS as described (4). The fraction of glucose derived from gluconeogenesis was measured according to methods developed by Chacko et al (5). Briefly, ^2H_2O enrichment was measured using gas chromatography combustion isotope ratio mass spectrometry (GC-IR-MS) and ^2H enrichment of glucose, excluding C2 determined with GC-MS.

Calculation of total glucose rate of appearance and glucose production

The total rate of appearance (Ra) of glucose into the systemic circulation was calculated under near steady state conditions by using the isotope dilution equation: Total Glucose Ra = [(*Ei/Ep*)-1] x F where Ei and Ep were the infusate and plasma [6,6-$^2H_2$] glucose, and *F* the rate of infusion of labeled glucose. In the fasting and insulin-stimulated steady states, HGP was calculated by subtracting the amount of labeled and unlabeled glucose from total glucose Ra:

HGP = Total Glucose Ra - *F* - *M*, where *M* was the average unlabeled glucose infusion rate to maintain euglycemia during the final 30 minutes of the clamp.

Measurement of gluconeogenesis and glycogenolysis

The fraction of glucose derived from gluconeogenesis (GNG) and glycogenolysis (GL) were determined using ^2H_2O and average ^2H enrichment of carbons 1,3,4,5, and 6 of glucose using the following formula.

Fractional GNG = $[(M+1)(^2H)(_{m/z170/169})/6]/^2H_2O$

GNG (mg/kgFFM/min) = Fractional GNG x Total Glucose Ra

GL mg/kgFFM/min) = EGP – GNG

where $[(M+1)(^2H)(_{m/z170/169})/6]$ is the M+1 average enrichment of deuterium measured using m/z 170/169, '6' is the number of ^2H labeling sites on the *m/z* 170/169 fragment of glucose. ^2H_2O is the deuterium enrichment in plasma water.

Measurement of insulin sensitivity

When HGP is completely suppressed during hyperinsulinemia, *M* reflects skeletal muscle glucose utilization and a direct measurement of peripheral insulin sensitivity. Homeostasis Model Assessment of Insulin Resistance (HOMA-IR) was calculated as fasting glucose (mmol/L) x fasting insulin (μIU/ml)/22.5. Metabolic clearance rate of insulin was calculated as insulin infusion rate divided by the difference between steady-state insulin and fasting plasma insulin concentration. Whole body insulin sensitivity was calculated using glucose and insulin concentrations from MMTT as:

$$\frac{10{,}000}{\sqrt{(\text{fasting glucose [mg/dL] x fasting insulin [}\mu\text{U/mL]})(\text{OGTT mean glucose [mg/dL] x OGTT mean insulin [}\mu\text{U/mL]})}}$$

Area under the curve (AUC) for glucose and insulin following MMTT were

calculated using the trapezoidal rule.

Associations of *CYP8B1* mutations with diabetes phenotypes in the T2D Knowledge Portal

Association analyses for *CYP8B1* mutant *R26X* were performed using the publicly available Type 2 Diabetes Knowledge Portal (http://www.type2diabetesgenetics.org). *R26X* was selected for association analysis because it was the most frequent CLOF mutation in our study cohort. Associations were calculated using the portal's Genetic Association Interactive Tool (GAIT), in which single-variant and gene-level association analysis can be conducted for any of 13 traits in 45,231 exomes from the AMP-T2D-GENES study. GAIT performs linear (for quantitative traits) or logistic (for dichotomous traits) regression of a phenotype on genotype, with options for stratifying samples by ancestry and conditioning on covariates for genetic ancestry and/or sample characteristics such as age, sex, and BMI. The genotypes and phenotypes in GAIT have been subjected to quality control as previously described (6). We used GAIT to test *R26X* for association with fasting insulin, adjusted for BMI, age, and sex.

In vitro skeletal muscle cell culture and transcript quantification

Human skeletal muscle cells (HSkMCs, adult) (non-immortalized human primary skeletal muscle myoblasts) were purchased from Cell Applications (cat# 150-05A). The culturing, sub-culturing, seeding and differentiation procedures were performed following manufacturer's instructions (https://www.sigmaaldrich.com/technical-documents/protocols/biology/human-skeletal-muscle-cells.html). Briefly, 6-well dishes were coated with collagen coating

solution (Sigma, 125-50) (1 ml/10cm^2 coating surface) by swirling the solution on the plate several times, and incubating for 30 minutes at 37°C. The coating was aspirated and the plates washed twice with Dulbecco's Phosphate Buffered Saline (Sigma, D8537). The adult HSkMC were seeded at 32,000 cells/cm^2 in Skeletal Muscle Cell Growth Medium (Sigma, 151-500). Cells were placed in a 37°C, 5% CO_2 humidified incubator overnight to induce differentiation the next day. For differentiation, Skeletal Muscle Cell Differentiation Medium (Sigma, 151D-250) was pre-equilibrated in a 37°C, 5% CO_2 humidified incubator for 2 hours. The Skeletal Muscle Cell Growth Medium (Sigma, 151-500) was removed from the cells by aspiration without allowing cells to dry during media changes. 1ml/5cm^2 Skeletal Muscle Cell Differentiation Medium (Sigma, 151D-250) was added to each well, and cells incubated in a 37°C, 5% CO_2 humidified incubator. Media were replaced with fresh Skeletal Muscle Cell Differentiation Medium every other day until multinuclear myotubes were formed (6-8 days). Cells from passages 4 to 9 were used. Robust differentiation was observed in all experiments as assessed by myotube formation.

For gene expression experiments, cells were seeded at 1.28x10^5/ml/well into 12-well plates and differentiation initiated the next day. After 8 days of differentiation, 50 μmol/L of cholic acid (CA):chenodeoxycholic acid (CDCA) mixtures or equal volume of dimethyl sulfoxide (DMSO) were added to the HSkMC differentiation medium supplemented with 0.5x Insulin-Transferrin-Selenium (ITS-G, Gibco). After 24 hours, cells were rinsed in PBS and stored at -80ºC. RNA was extracted using the RNeasy Plus Mini Kit (Qiagen), and cDNA was generated by using random primers (Promega) and SuperScript™ III Reverse Transcriptase (Invitrogen). Quantitative real-time PCR was performed using SYBR Select Master Mix (Applied Biosystems) in a QuantStudio 6 Flex Real-Time PCR System (Applied

Biosystems). Reactions were performed in triplicates with following primers: *hIRB* fwd: 5'CCCCAGAAAAACCTCTTCAGG, *hIRB* rev: 5'GTCACATTCCCAACATCGCC; *hFOXO1* fwd: 5'TGGACATGCTCAGCAGACATC, *hFOXO1* rev: 5'TTGGGTCAGGCGGTTCA; *hGAPDH* fwd: 5'GAAGGTGAAGGTCGGAGT, *hGAPDH* rev: 5'CATGGGTGGAATCATATTGGAA. Quantitative PCR analyses were performed by the comparative Ct method, using glyceraldehyde-3-phosphate dehydrogenase (*hGAPDH*) as internal controls. For the gene expression and glucose uptake assays, each N was from one well of a 6-well dish. For western blots, each N was combined from three wells of a 6-well dish in order to obtain sufficient protein for the AKT and FOXO1 immunoblots. All N's are shown in all figures.

Skeletal muscle cell phospho-AKT and phospho-FOXO1 quantification

To quantify AKT and FOXO1 phosphorylation in the insulin-stimulated state, differentiated HSkMCs were treated with 50 μmol/L CA:CDCA mixtures or other bile acid mixes and concentrations as shown in the figures, in HSkMC starvation medium for 24 hours. Controls were treated with equal volume of DMSO in HSkMC starvation medium. The cells were then stimulated in fresh starvation medium with or without 100 nmol/L of insulin (Humalog 100 units/ml, Eli Lilly) for 15 minutes. At the end of insulin stimulation, cells were rinsed and harvested in cold PBS, and homogenized in lysis buffer (50 mmol/L HEPES pH 7.4, 1% Triton-X-100, 0.1 mol/L NaF, 10 mmol/L EDTA, 50 mmol/L NaCl, 10 mmol/L orthovanadate, 0.1% SDS and 1x protease inhibitor). Proteins were resolved on 7.5% polyacrylamide gels, transferred to PVDF membranes, and probed with anti-phospho-AKT (#9271, Cell Signaling), anti-AKT (#2967, Cell Signaling), anti-phospho-FOXO1 (Ser256) (#9461,

Cell Signaling), or anti-FOXO1 (#2880, Cell Signaling) antibodies. Blots were developed by chemiluminescence (Bio-Rad). Relative densities were calculated using ImageJ.

Skeletal muscle cell glucose uptake

For glucose uptake experiments, differentiated human fetal skeletal muscle cell (Cell Applications #150-05f) myotubes were treated with carrier and control BA mix as above. Cells were washed with KRBH buffer (10 mM sodium phosphate, 1 mM $MgSO_4$, 1 mM $CaCl_2$, 136 mM NaCl, 4.7 mM KCl, 10 mM HEPES, pH 7.6) and incubated ±100 nM insulin for 1 hour in buffer containing 10 μM 2-NBDG (2-(N-(7-Nitrobenz-2-oxa-1,3-diazol-4-yl)amino)-2-deoxyglucose; N13195, ThermoFisher Scientific). Cells were washed and glucose uptake quantified using a fluorescence plate reader (POLARstar Omega, BMG Labtech, Excitation = 465 nm, Emission = 530 nm). Values were normalized to protein content and expressed relative to uptake of control treatment myotubes in the absence of insulin.

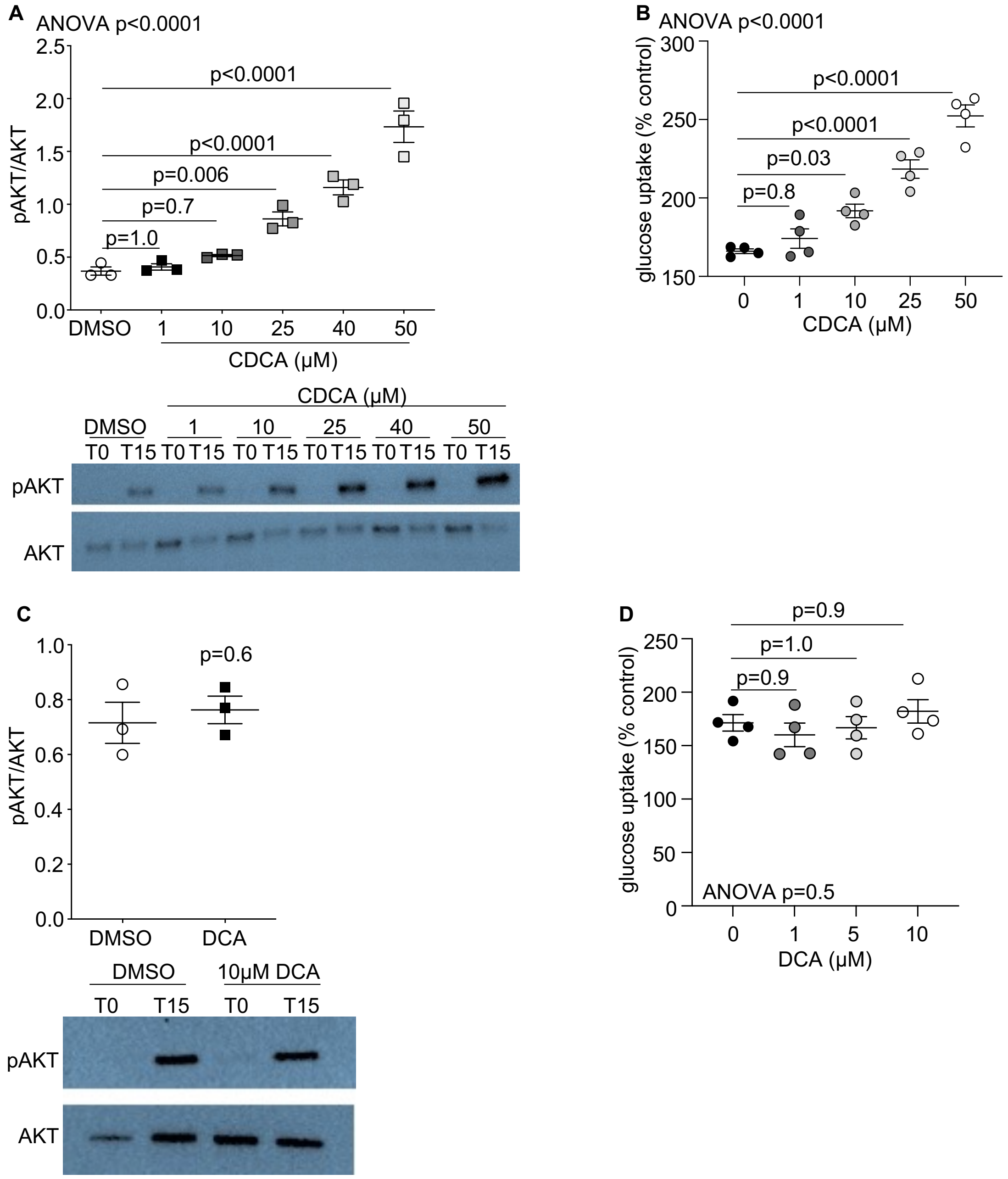

Supplemental Figure 1. Dose response to CDCA and response to deoxycholic acid in skeletal muscle cells. The dose response of CDCA on **(A)** muscle cell insulin signaling, assessed by quantifying levels of phosphorylated AKT, and **(B)** muscle cell glucose uptake. The impact of deoxycholic acid (DCA) on primary skeletal muscle cell **(C)** insulin signaling, assessed by quantifying levels of phosphorylated AKT, and **(D)** glucose uptake. CDCA=chenodeoxycholic acid. Data are shown as average±standard error. Data in (A) and (C) are quantified 15 minutes after insulin

stimulation, and data in (B) and (D) are quantified 1 hour after insulin stimulation. Data in (A), (B) and (D) are analyzed using One-way ANOVA followed by Tukey's post-tests. Data in (C) are normally distributed and analyzed using unpaired *t*-tests.

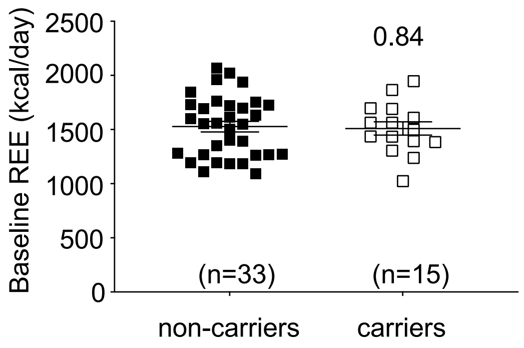

Supplemental Figure 2. Baseline resting energy expenditure (REE) is not significantly different between human *CYP8B1* mutation carriers and non-carrier controls. Data are shown as average ± standard error. Data were normally distributed and analyzed using unpaired *t*-tests.

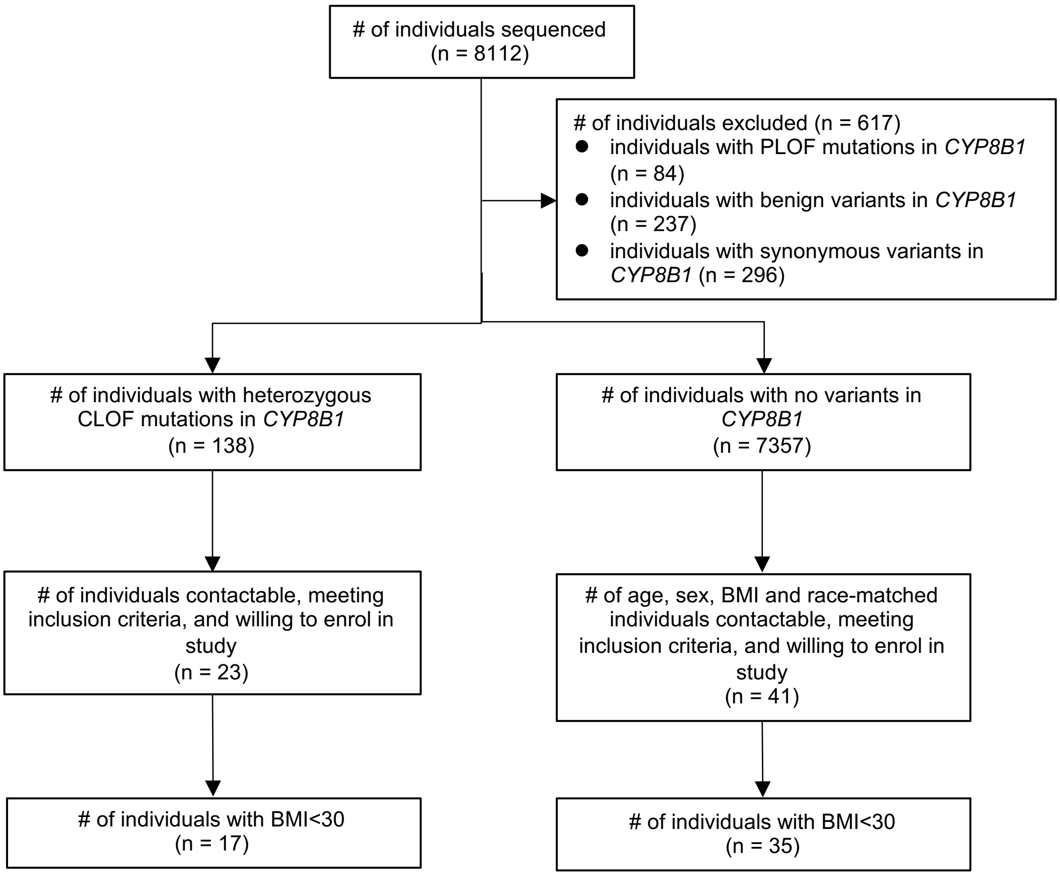

Supplemental Figure 3. Flow chart of subject recruitment for the study.

Supplemental Table 1. Predicted function and assay-verified *in vitro* function of all *CYP8B1* variants.

Amino Acid Change	Nucleotide Change	Mutation Type	PolyPhen-2 Prediction	SIFT Prediction	In vitro Function (% of wild-type)	Mutation Category
Q372K	C1114A	missense	BN	TL	143.0	BN
D418G	A1253G	missense	PS	DM	121.9	BN
V402I	G1204A	missense	BN	TL	120.9	BN
R306Q	G917A	missense	BN	TL	119.6	BN
K238R	A713G	missense	BN	TL	118.1	BN
H124Y	C370T	missense	PS	DM	112.4	BN
L357F	C1069T	missense	BN	TL	111.8	BN
E193Q	G577C	missense	BN	TL	110.2	BN
E322Q	G964C	missense	PS	TL	110.2	BN
E121K	G361A	missense	BN	TL	109.3	BN
H378Q	T1134G	missense	BN	TL	106.5	BN

Supplemental Table 1. (Continued.)

Amino Acid Change	Nucleotide Change	Mutation Type	PolyPhen-2 Prediction	SIFT Prediction	In vitro Function (% of wild-type)	Mutation Category
A270V	C809T	missense	BN	TL	105.8	BN
V437I	G1309A	missense	BN	TL	104.9	BN
T470I	C1409T	missense	BN	TL	103.1	BN
S245I	G734T	missense	BN	TL	102.7	BN
M239I	G717C	missense	BN	TL	101.2	BN
S488N	G1463A	missense	BN	TL	100.8	BN
P88S	C262T	missense	BN	TL	98.3	BN
D195E	C585A	missense	PD	TL	98.1	BN
A10T	G28A	missense	BN	TL	96.8	BN
V80I	G238A	missense	PS	TL	96.2	BN
S269T	T805A	missense	BN	TL	94.9	BN

Supplemental Table 1. (Continued.)

Amino Acid Change	Nucleotide Change	Mutation Type	PolyPhen-2 Prediction	SIFT Prediction	In vitro Function (% of wild-type)	Mutation Category
C163S	T487A	missense	BN	TL	94.6	BN
R171H	G512A	missense	BN	TL	91.6	BN
P468A	C1402G	missense	PS	TL	88.2	BN
R95K	G284A	missense	BN	TL	87.0	BN
D406N	G1216A	missense	BN	TL	86.1	BN
L316V	C946G	missense	BN	TL	84.2	PLOF
D195N	G583A	missense	PS	TL	82.4	PLOF
T153M	C458T	missense	PS, BN	TL	80.9	PLOF
H301Y	C901T	missense	BN	TL	76.1	PLOF
D160Y	G478T	missense	PD, PS	DM	74.7	PLOF
R28H	G83A	missense	PD	DM	74.1	PLOF

Supplemental Table 1. (Continued.)

Amino Acid Change	Nucleotide Change	Mutation Type	PolyPhen-2 Prediction	SIFT Prediction	In vitro Function (% of wild-type)	Mutation Category
Q233R	A698G	missense	BN	TL	73.7	PLOF
K330Q	A988C	missense	BN	TL	71.7	PLOF
E55G	A164G	missense	BN	TL	71.6	PLOF
R171C	C511T	missense	PS	DM	70.9	PLOF
K300N	G900C	missense	PD	DM	70.5	PLOF
E310V	A929T	missense	BN	DM	70.4	PLOF
R59C	C175T	missense	PD	DM	70.0	PLOF
I123L	A367T	missense	BN	TL	69.6	PLOF
T430A	A1288G	missense	BN	TL	68.7	PLOF
V242M	G724A	missense	PD	TL	66.7	PLOF
M53T	T158C	missense	BN	TL	63.5	PLOF

Supplemental Table 1. (Continued.)

Amino Acid Change	Nucleotide Change	Mutation Type	PolyPhen-2 Prediction	SIFT Prediction	In vitro Function (% of wild-type)	Mutation Category
G187S	G559A	missense	PD	DM	63.0	PLOF
R492H	G1475A	missense	PD, PS	TL	62.9	PLOF
R207C	C619T	missense	PD	TL	59.9	PLOF
V339L	G1015C	missense	BN	TL	59.8	PLOF
E247 del-nt.GAG739-741 del	del 739-741(GAG)	deletion	-	-	55.8	PLOF
K192E	A574G	missense	BN	TL	54.5	PLOF
R207L	G620T	missense	PS	TL	52.4	PLOF
P398L	C1193T	missense	PS	DM	52.0	PLOF
F419L	T1255C	missense	PD	DM	48.4	PLOF
R28C	C82T	missense	PD	DM	48.2	PLOF
D341E	C1023G	missense	PS	TL	47.4	PLOF

Supplemental Table 1. (Continued.)

Amino Acid Change	Nucleotide Change	Mutation Type	PolyPhen-2 Prediction	SIFT Prediction	In vitro Function (% of wild-type)	Mutation Category
R496C	C1486T	missense	PD	DM	45.9	PLOF
V70A	T209C	missense	PD	DM	42.8	PLOF
V343M	G1027A	missense	PD	DM	41.6	PLOF
L97V	C289G	missense	PD	DM	40.0	PLOF
R207H	G620A	missense	PD, PS	TL	38.5	PLOF
R494H	G1481T	missense	PD	DM	38.0	PLOF
D490N	G1468A	missense	PD	DM	35.0	PLOF
R234P	G701C	missense	BN	TL	34.0	PLOF
T287M	C860T	missense	PD, PS	TL	34.0	PLOF
R415Q	G1244A	missense	PS, BN	TL	31.8	PLOF
R494C	C1480T	missense	PD, PS	DM	31.5	PLOF

Supplemental Table 1. (Continued.)

Amino Acid Change	Nucleotide Change	Mutation Type	PolyPhen-2 Prediction	SIFT Prediction	In vitro Function (% of wild-type)	Mutation Category
R496H	G1487A	missense	PD, PS	DM	30.5	PLOF
Y102C	A305G	missense	PD	DM	29.0	PLOF
L108P	T323C	missense	PS	TL	28.6	PLOF
R377H	G1130A	missense	PD	DM	26.2	PLOF
F186L	T556C	missense	PD	DM	24.6	PLOF
D119N	G355A	missense	BN	TL	23.1	PLOF
V475D	T1424A	missense	PD, PS	DM	19.3	PLOF
R50Q	G149A	missense	PD	TL	19.2	PLOF
F170S	T509C	missense	PD	DM	18.7	PLOF
F86S	T257C	missense	PD	DM	18.3	PLOF
R26Q	G77A	missense	PD	DM	15.6	PLOF

Supplemental Table 1. (Continued.)

Amino Acid Change	Nucleotide Change	Mutation Type	PolyPhen-2 Prediction	SIFT Prediction	In vitro Function (% of wild-type)	Mutation Category
L220P (0.00055)	T659C	missense	PD	DM	10.4	CLOF
E311D (0.00006)	A933T	missense	PD	DM	6.4	CLOF
A103E (0.00006)	C308A	missense	PD	DM	5.6	CLOF
S342R (0.00006)	A1024C	missense	PD	DM	2.9	CLOF
R407H (0.00006)	G1220A	missense	PD	DM	1.7	CLOF
E311G (0.00006)	A932G	missense	PD	DM	1.5	CLOF
L147P (0.00006)	T440C	missense	PD	DM	0.8	CLOF
R479C (0.00049)	C1435T	missense	PD	DM	0.8	CLOF
Q486K (0.00006)	C1456A	missense	PD, PS	TL	0.8	CLOF
R26X (0.0049)	C76T	nonsense	-	-	0.6	CLOF
R207P (0.00006)	G620C	missense	PD	TL	0.3	CLOF

Supplemental Table 1. (Continued.)

Amino Acid Change	Nucleotide Change	Mutation Type	PolyPhen-2 Prediction	SIFT Prediction	In vitro Function (% of wild-type)	Mutation Category
E345 del-nt.GAG1033-1035 del (0.00012)	del 1033-1035(GAG)	deletion	-	-	0.2	CLOF
Q194X (0.00006)	C580T	nonsense	-	-	0.1	CLOF
L72P (0.00025)	T215C	missense	PD	DM	0.1	CLOF
S438fs-nt.C1313 del (0.00006)	C1313del	frame shift	-	-	0.0	CLOF
R95fs-nt.A285 del (0.00006)	A285del	frame shift	-	-	0.0	CLOF
G288R (0.00086)	G862C	missense	PD	DM	0.0	CLOF
M53fs-nt.AT157-158 del (0.00006)	del 157-158(AT)-fs	frame shift	-	-	0.0	CLOF
G434R (0.00006)	G1300C	missense	PD	DM	0.0	CLOF
K300X (0.00006)	A898T	nonsense	-	-	0.0	CLOF
R349Q (0.00018)	G1046A	missense	PD	DM	0.0	CLOF
R349W (0.00006)	C1045T	missense	PD	DM	0.0	CLOF

Supplemental Table 1. (Continued.)

Amino Acid Change	Nucleotide Change	Mutation Type	PolyPhen-2 Prediction	SIFT Prediction	In vitro Function (% of wild-type)	Mutation Category
L298H (0.00006)	T893A	missense	PD	DM	0.0	CLOF

BN=benign; PS=possibly damaging; PD=probably damaging; TL=tolerated; DM=damaging; PLOF=partial loss of function; CLOF=complete loss of function. Where there are two predictions by PolyPhen-2, the former is from the HumDiv-trained PolyPhen-2 model, and the latter from the HumVar-trained PolyPhen-2 model. For the other PolyPhen-2 predictions, the two models give the same result. Minor allele frequencies are given in parentheses for the complete loss of function mutations.

Supplemental Table 2. Plasma bile acid profile of heterozygous *CYP8B1* mutation carriers and controls.

nmol/L	non-carrier controls	mutation carriers	p-value
7α-HCO*	5.91 (7.40)	11.77 (16.42)	0.02
7α,12α-diHCO*	0.37 (0.36)	0.34 (0.40)	0.30
product:substrate†	0.060 (0.032)	0.022 (0.009)	<0.0001
CA	54.53 (59.92)	31.82 (17.51)	0.01
GCA	255.61 (365.14)	100.95 (102.88)	0.01
TCA	32.80 (63.96)	19.76 (22.94)	0.048
DCA	548.83 (548.68)	284.53 (412.09)	0.003
GDCA	204.25 (386.41)	118.01 (110.21)	0.02
TDCA	31.35 (40.79)	16.22 (20.34)	0.04
CDCA	362.50 (418.03)	314.63 (268.72)	0.63
GCDCA	1311.59 (2240.06)	1369.57 (1274.29)	0.74
TCDCA	111.60 (174.38)	109.54 (167.86)	0.99
LCA	31.18 (22.47)	35.96 (47.36)	0.82
GLCA	16.21 (20.83)	15.62 (23.73)	0.48
TLCA	2.05 (3.20)	2.49 (5.58)	0.78
UDCA	71.65 (99.08)	67.84 (60.13)	0.89
GUDCA	156.64 (351.28)	207.19 (336.83)	0.57
TUDCA and THDCA	11.02 (11.23)	9.84 (11.58)	0.95
HDCA	4.78 (5.51)	4.85 (8.18)	0.61

Supplemental Table 2. (Continued.)

nmol/L	non-carrier controls	mutation carriers	p-value
GHDCA	0.86 (0.94)	2.15 (2.28)	0.04
β-MCA	1.28 (2.32)	1.88 (2.47)	0.10
GHCA	27.94 (26.23)	43.32 (66.44)	0.28
THCA	2.73 (3.67)	6.16 (8.32)	0.29
Total bile acids	18978.23 (7570.53)	19205.83 (6029.90)	0.85
Total 12α bile acids	1449.93 (1442.51)	692.48 (577.14)	0.0002
Total non-12α bile acids	2936.26 (3061.75)	2599.62 (1935.91)	0.89
12α:non-12α ratio[†]	0.57 (0.46)	0.29 (0.37)	0.003
CA:CDCA ratio[†]	0.20 (0.21)	0.10 (0.10)	0.01
(T/G)CDCA:12α+non-12α ratio[†]	0.46 (0.17)	0.60 (0.16)	0.03

7α-HCO=7α-hydroxy-4-cholesten-3-one (substrate); 7α,12α-diHCO=7α,12α-dihydroxy-4-cholesten-3-one (product). CA=cholic acid; GCA=glycocholic acid; TCA=taurocholic acid; DCA=deoxycholic acid; GDCA=glycodeoxycholic acid; TDCA=taurodeoxycholic acid; CDCA=chenodeoxycholic acid; GCDCA=glycochenodeoxycholic acid; TCDCA=taurochenodeoxycholic acid; LCA=lithocholic acid; GLCA=glycolithocholic acid; TLCA=taurolithocholic acid; UDCA=ursodeoxycholic acid; GUDCA=glycoursodeoxycholic acid; TUDCA=tauroursodeoxycholic acid; HDCA=hyodeoxycholic acid; GHDCA=glycohyodeoxycholic acid; THDCA=taurohyodeoxycholic acid; β-MCA=β-muricholic acid; GHCA=glycohyocholic acid; THCA=taurohyocholic acid;

(T/G)CDCA=TCDCA+GCDCA+CDCA. * The unit of concentration is nmol/ml. † The ratio has no units.

References

1. Foong AW, et al. Rationale and methodology for a population-based study of eye diseases in Malay people: The Singapore Malay eye study (SiMES). *Ophthalmic Epidemiol*. 2007;14(1):25-35.

2. Tan KHX, et al. Cohort Profile: The Singapore Multi-Ethnic Cohort (MEC) study. *Int J Epidemiol*. 2018;47(3):699-699j.

3. Win AM, et al. Patterns of physical activity and sedentary behavior in a representative sample of a multi-ethnic South-East Asian population: a cross-sectional study. *BMC Public Health*. 2015;15:318-329.

4. Tan HC, et al. Comprehensive assessment of insulin resistance in non-obese Asian Indian and Chinese men. *J Diabetes Investig*. 2018;9(6):1296-1303.

5. Chacko SK, et al. Measurement of gluconeogenesis using glucose fragments and mass spectrometry after ingestion of deuterium oxide. *J Appl Physiol (1985)*. 2008;104(4):944-951.

6. Flannick J, et al. Exome sequencing of 20,791 cases of type 2 diabetes and 24,440 controls. *Nature*. 2019;570(7759):71-76.

Full un-edited blots for Figure 1

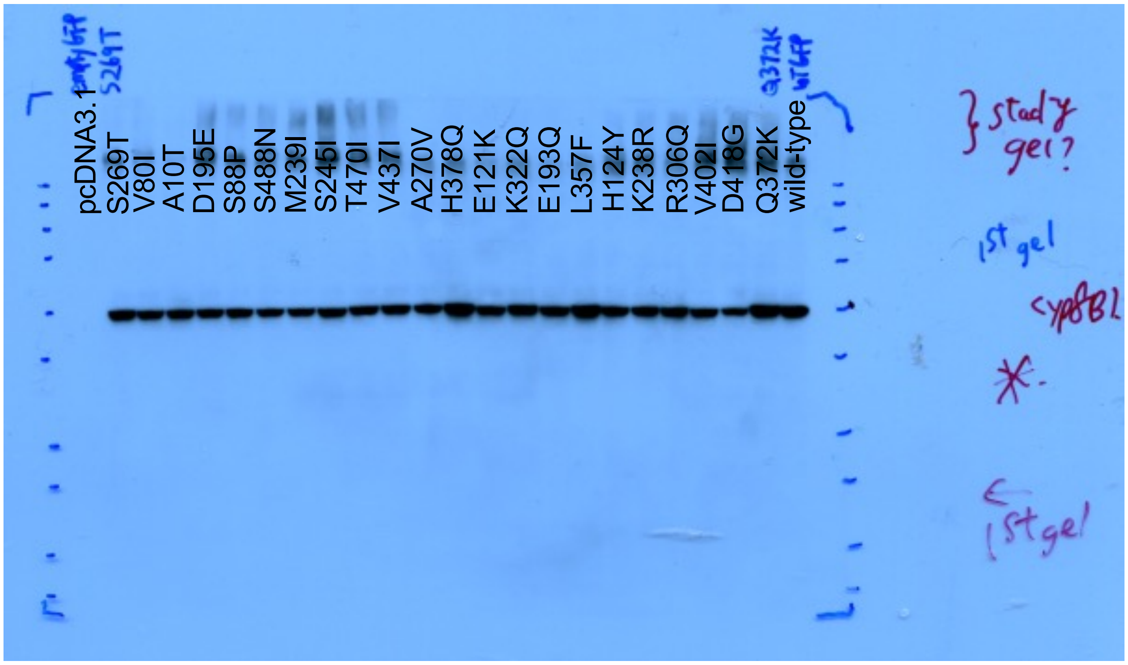

CYP8B1

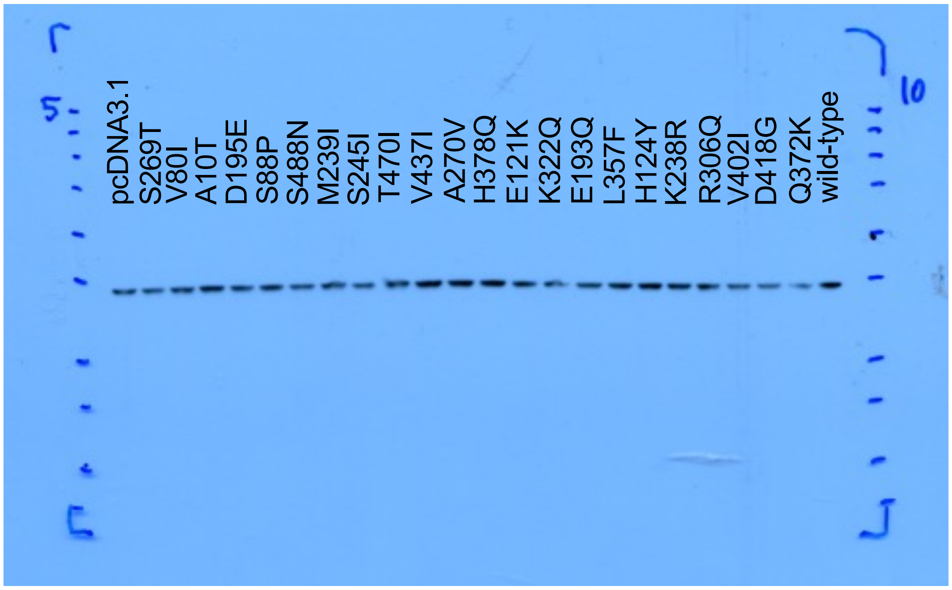

GAPDH

Gel set #1

Full un-edited blots for Figure 1

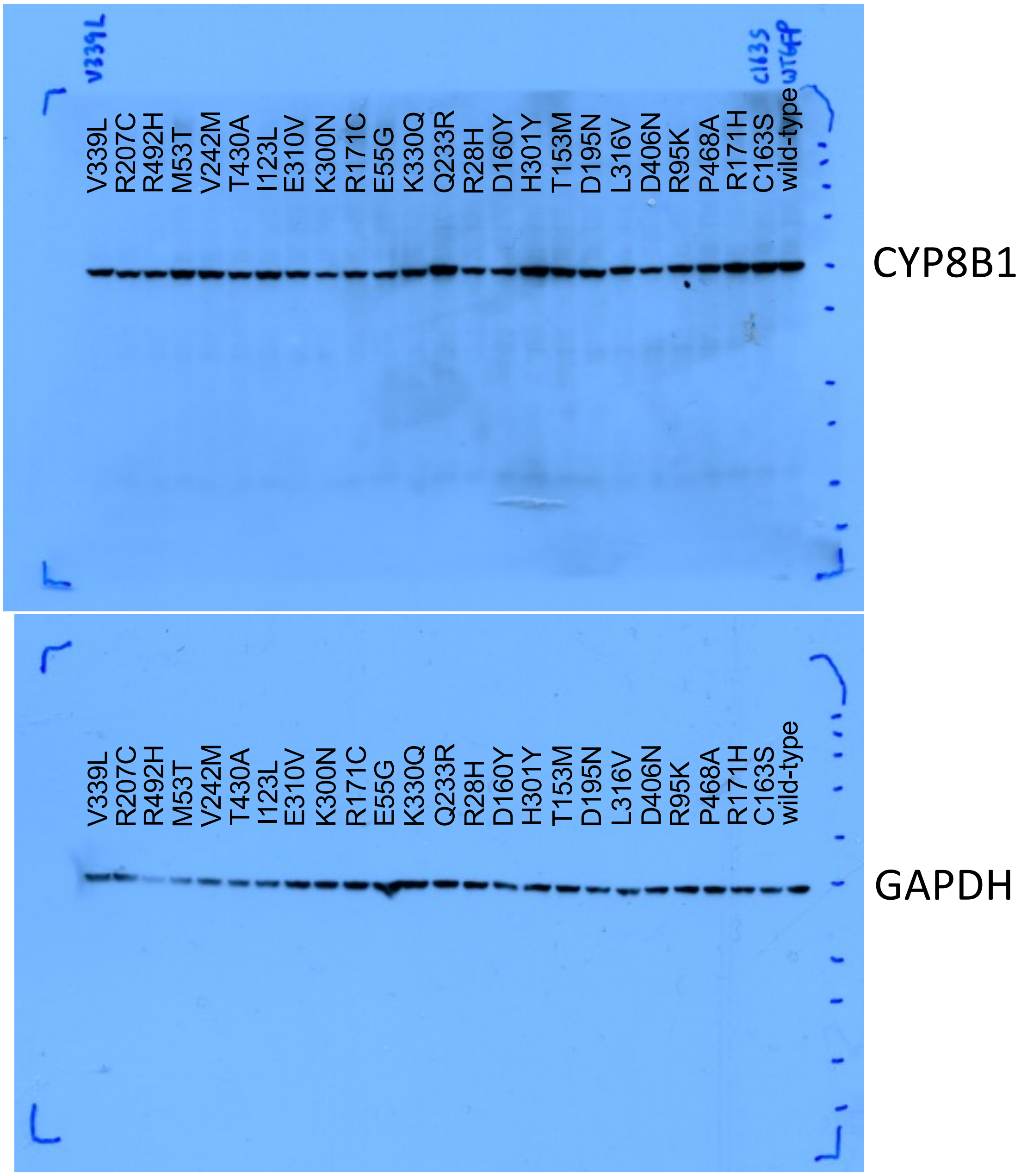

Gel set #2

Full un-edited blots for Figure 1

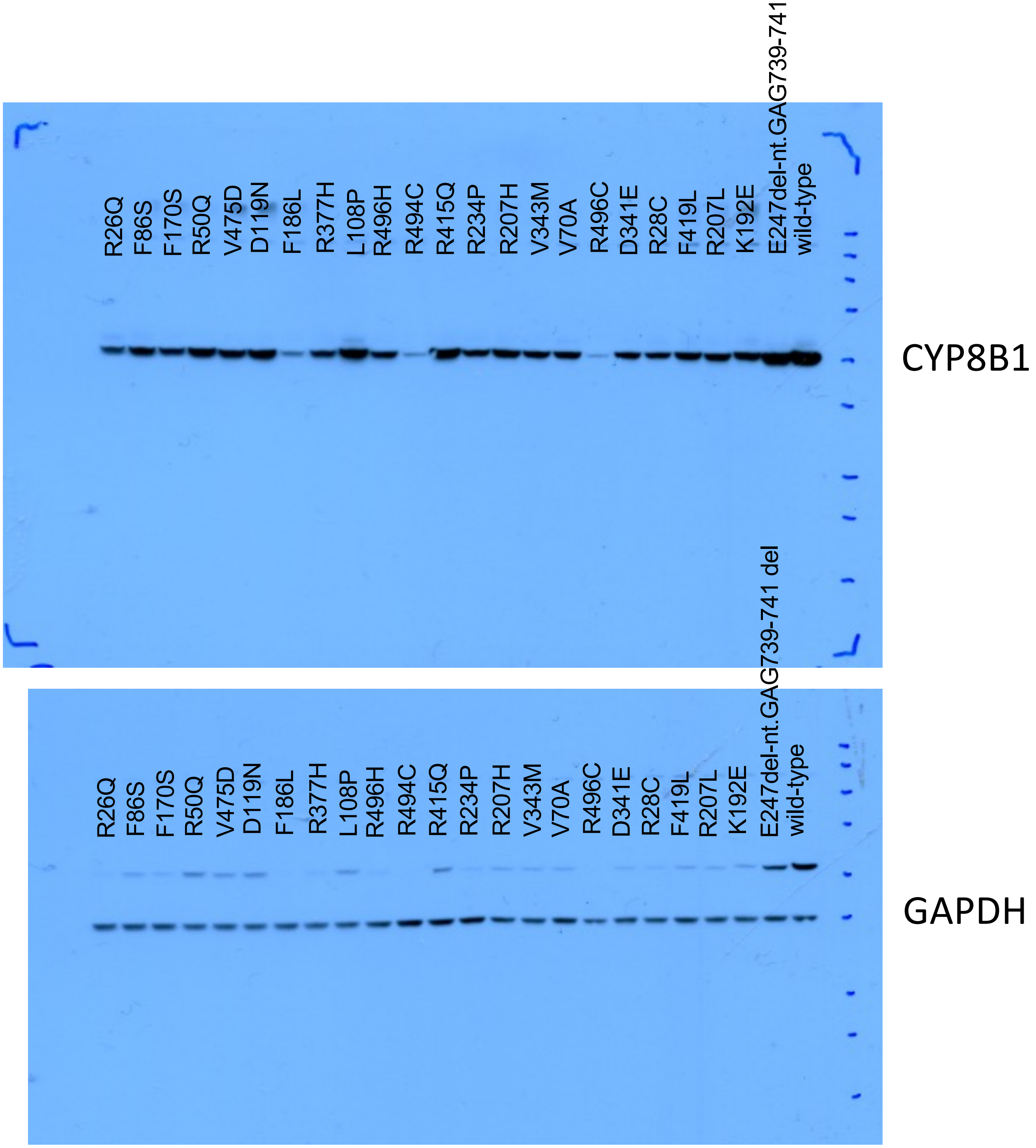

Gel set #3

Full un-edited blots for Figure 1

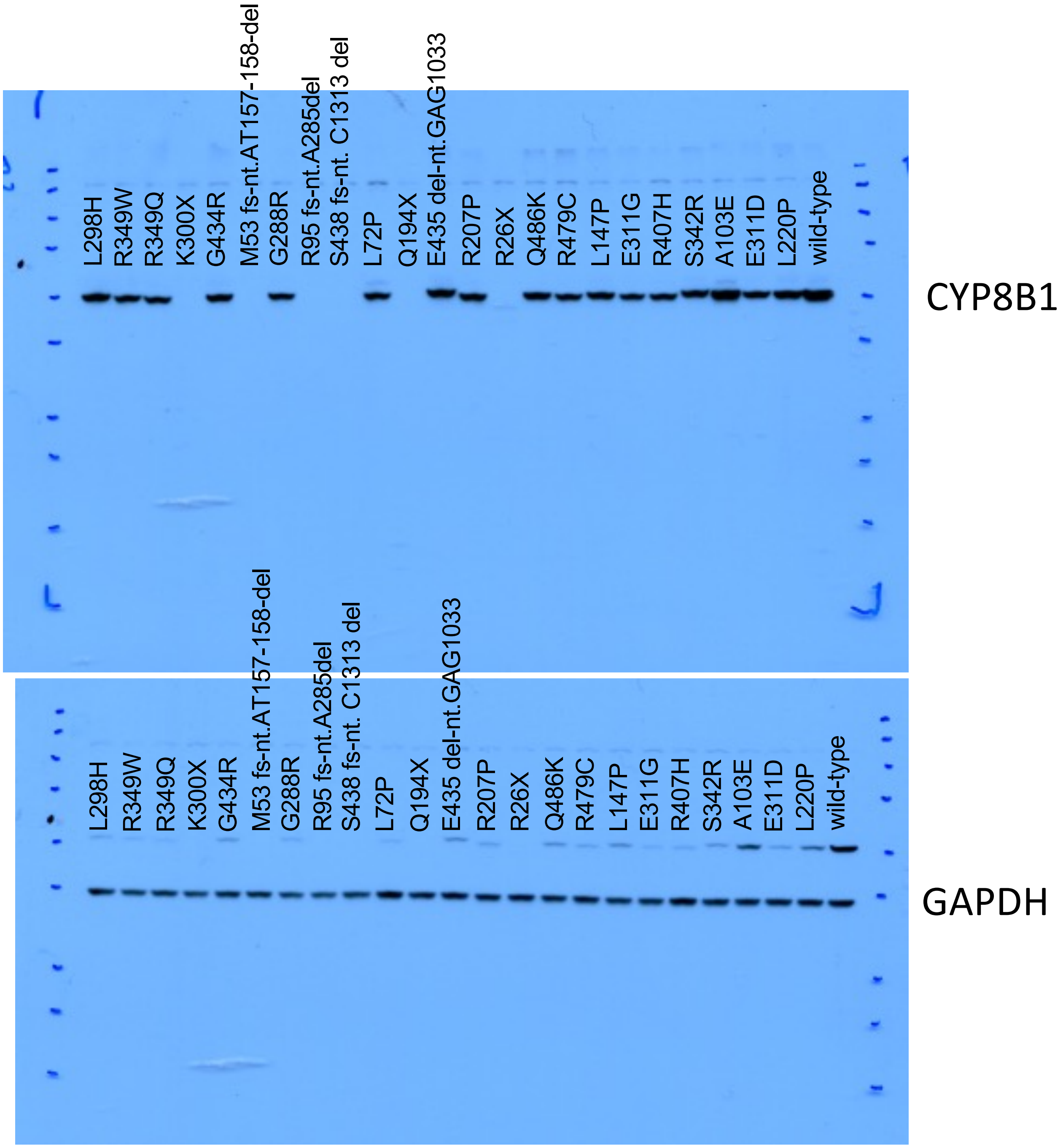

Gel set #4

Full un-edited blots for Figure 5C

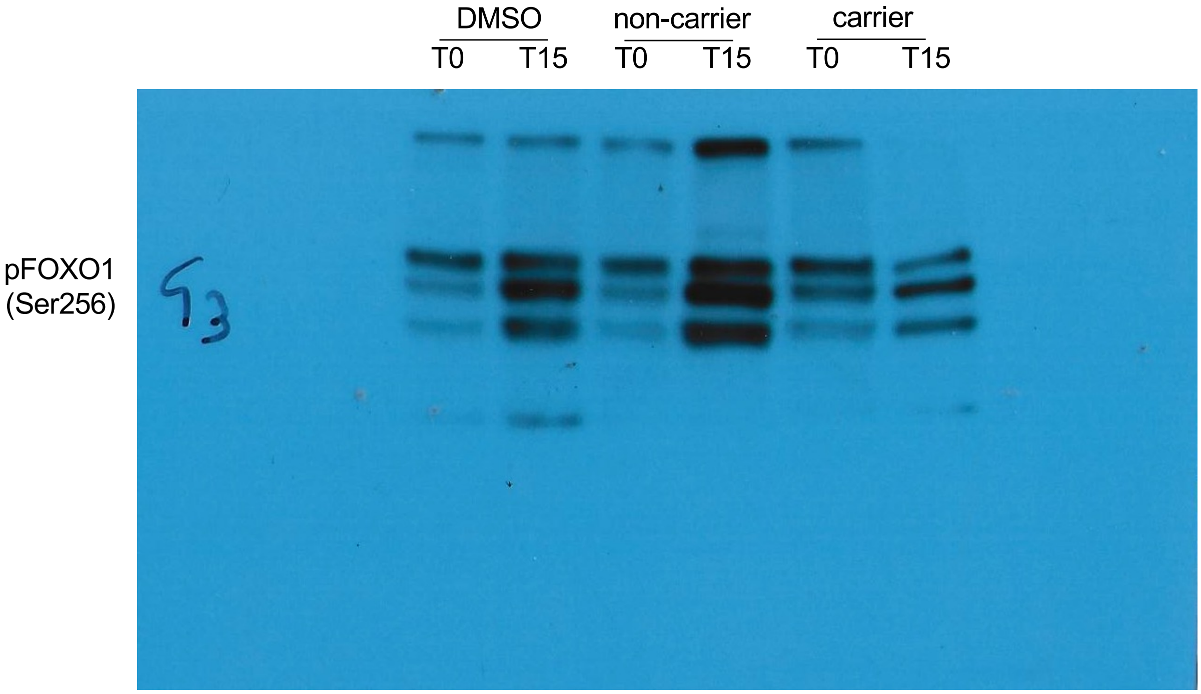

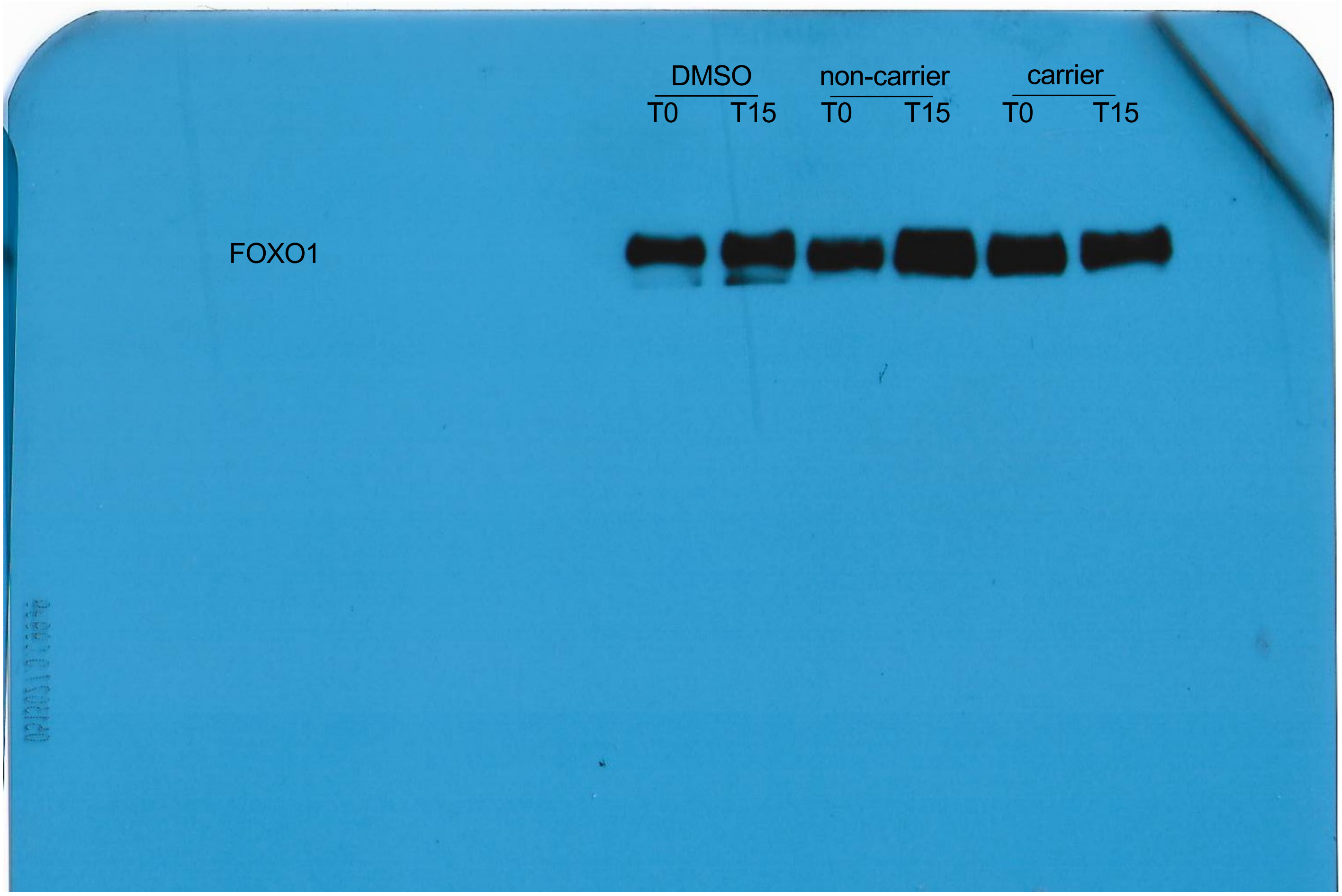

Full un-edited blots for Figure 5D

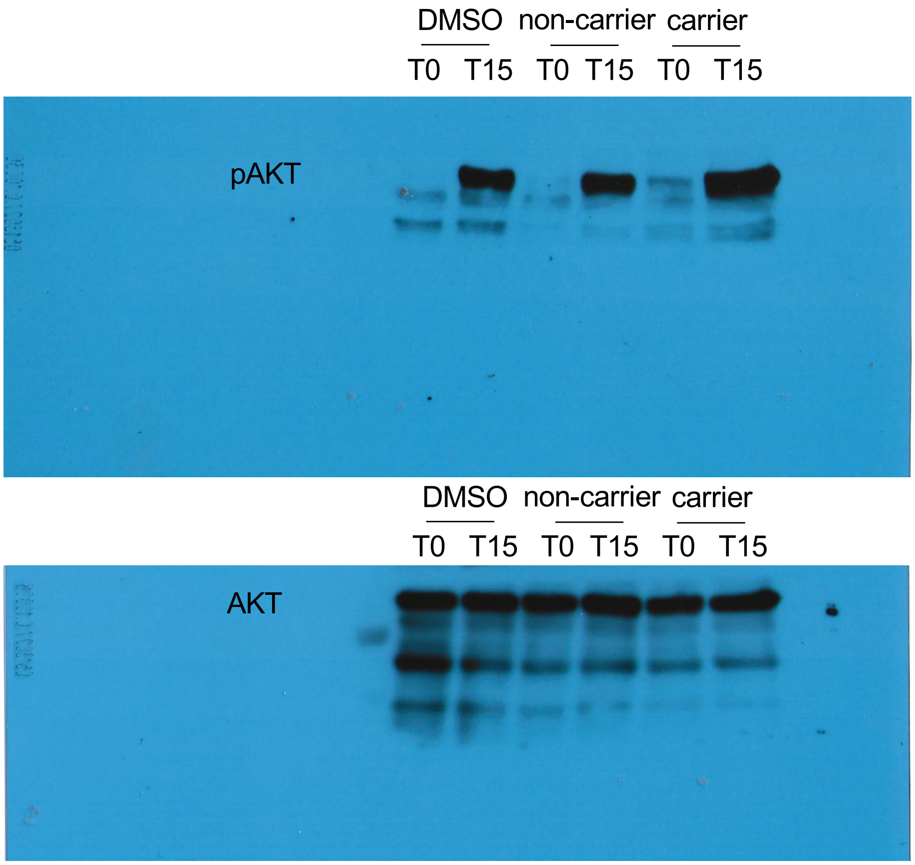

Full un-edited blots for Figure 5F

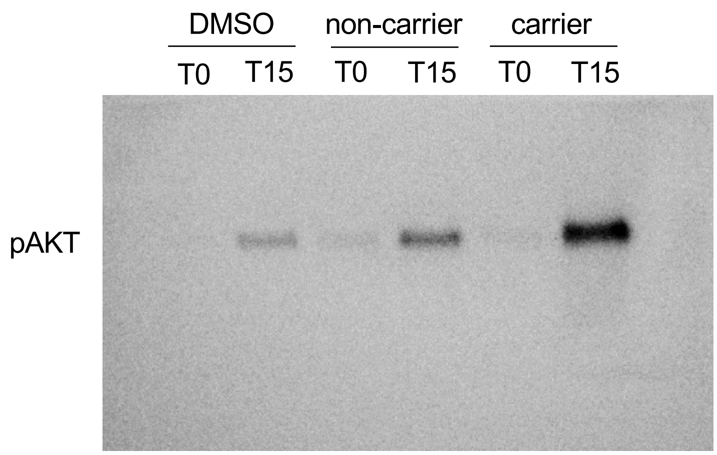

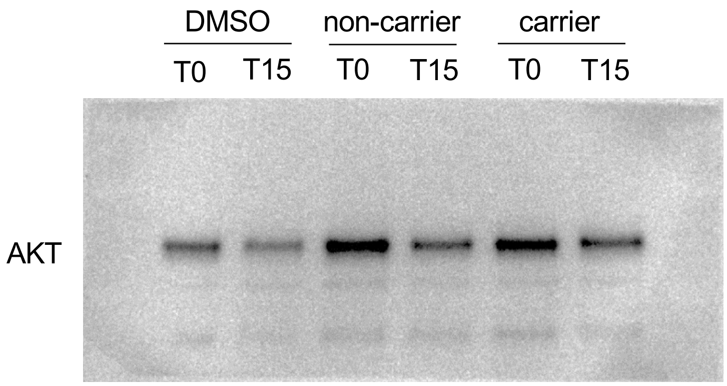

Full un-edited blots for Figure 6A

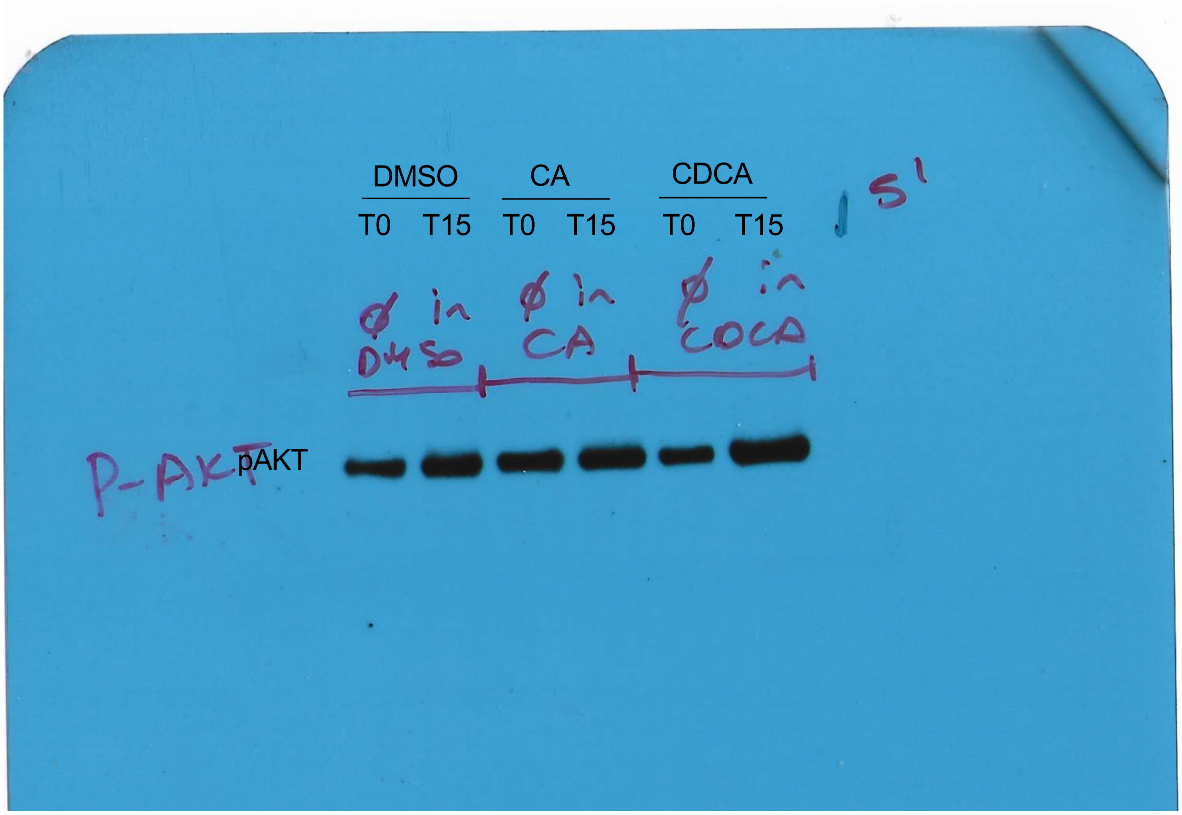

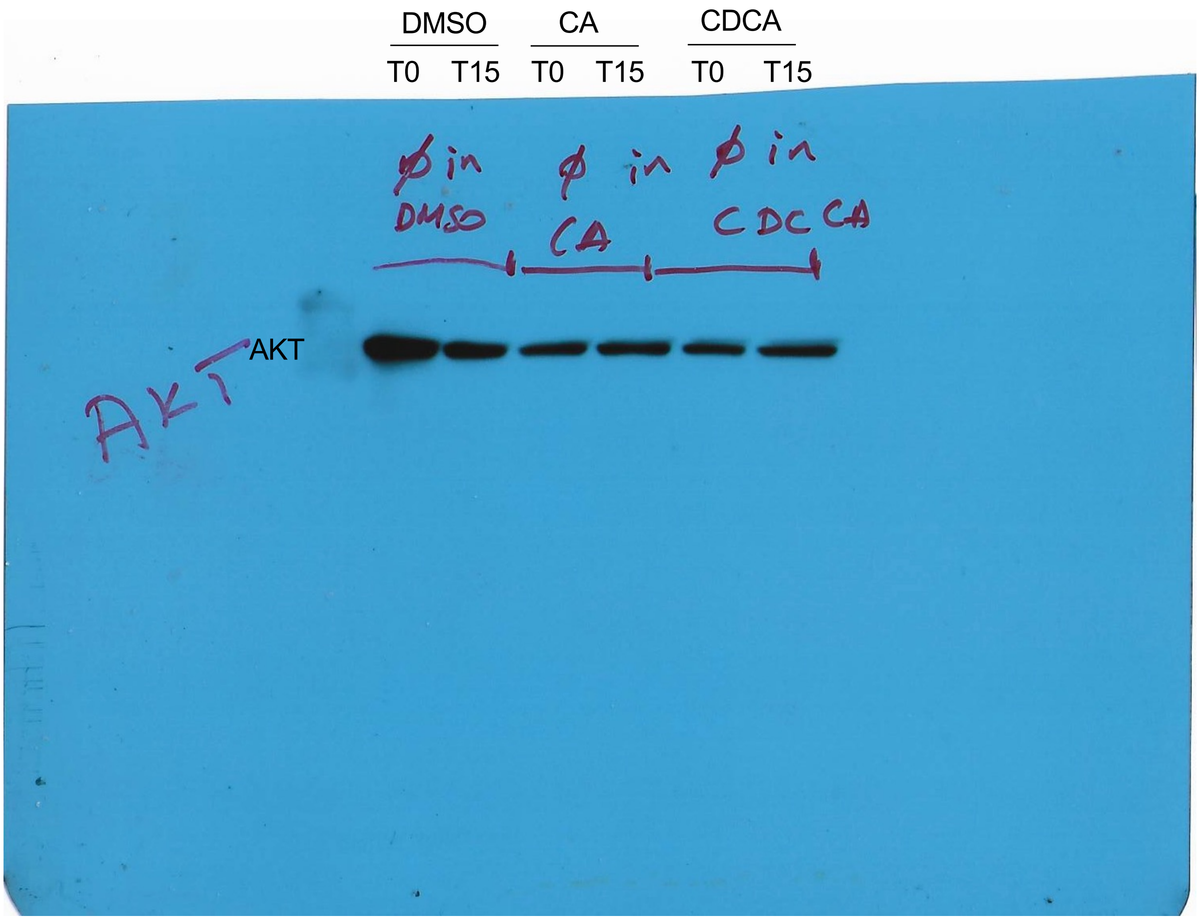

Full un-edited blots for Figure 6E

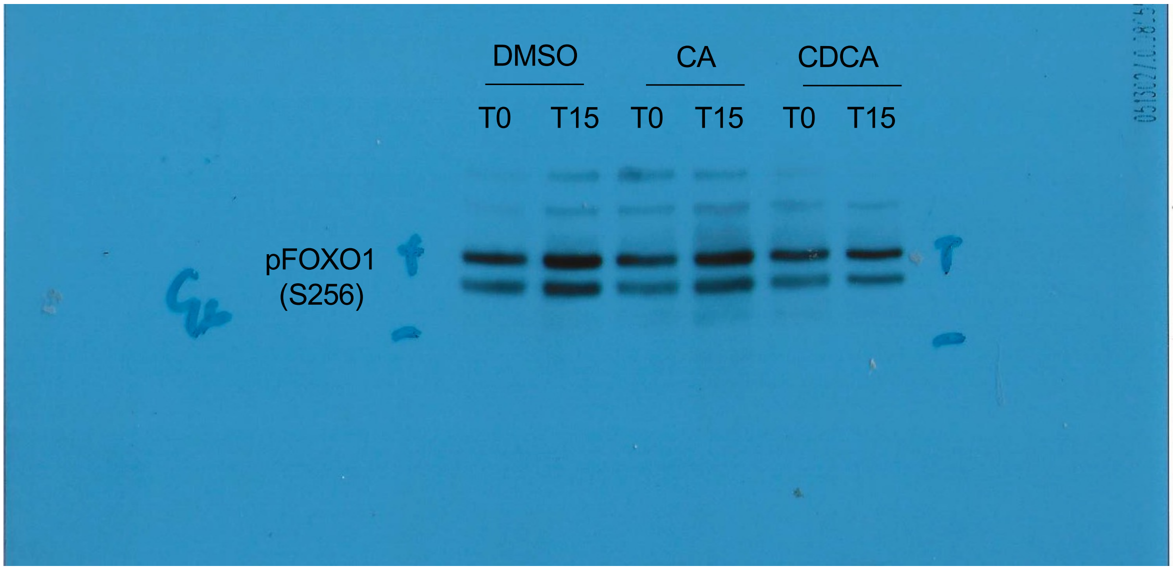

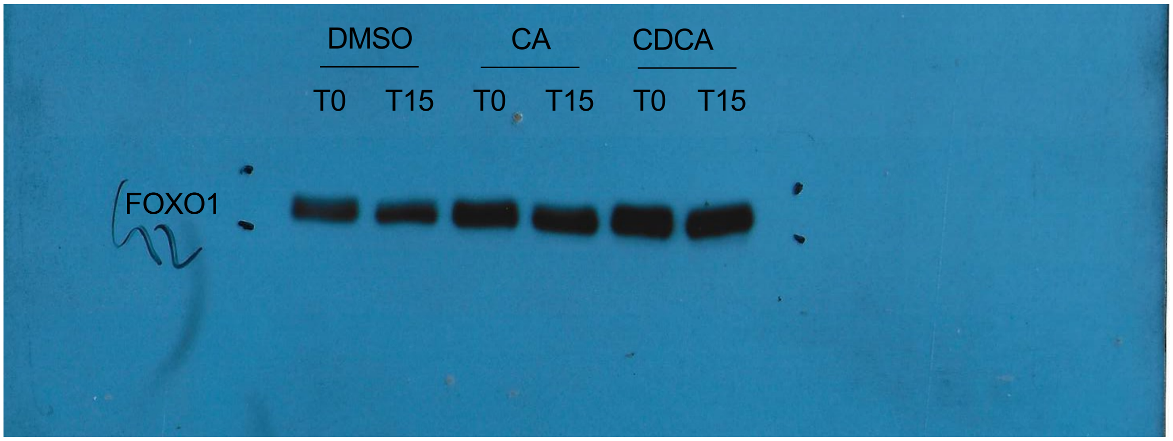

Full un-edited blots for Figure 6F

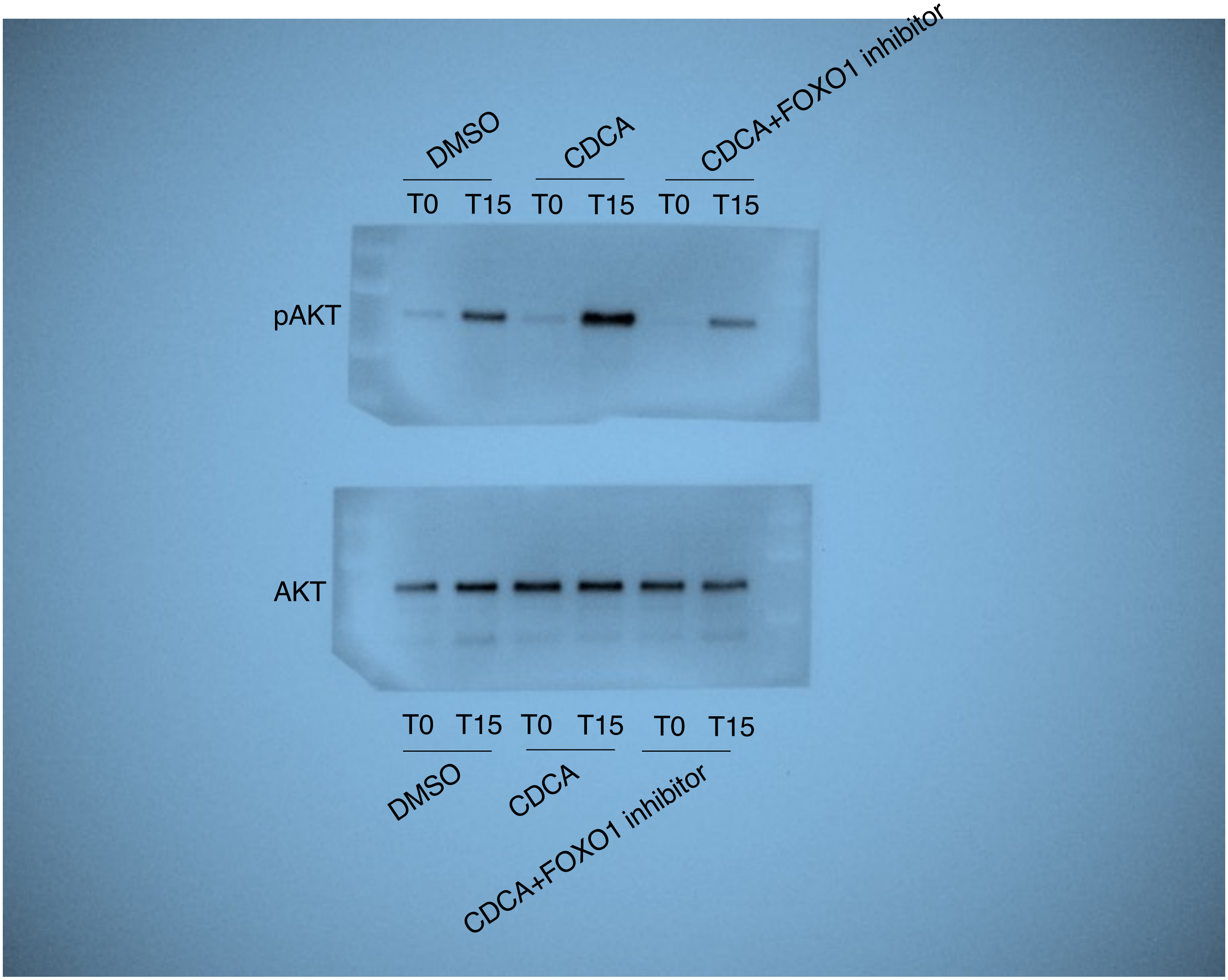

Full un-edited blots for Supplemental Figure 1A

pAKT

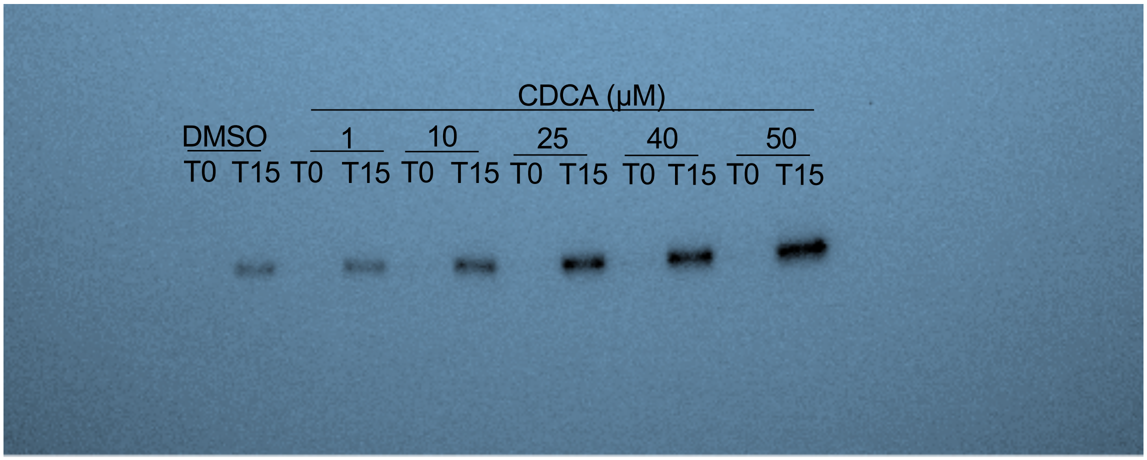

AKT

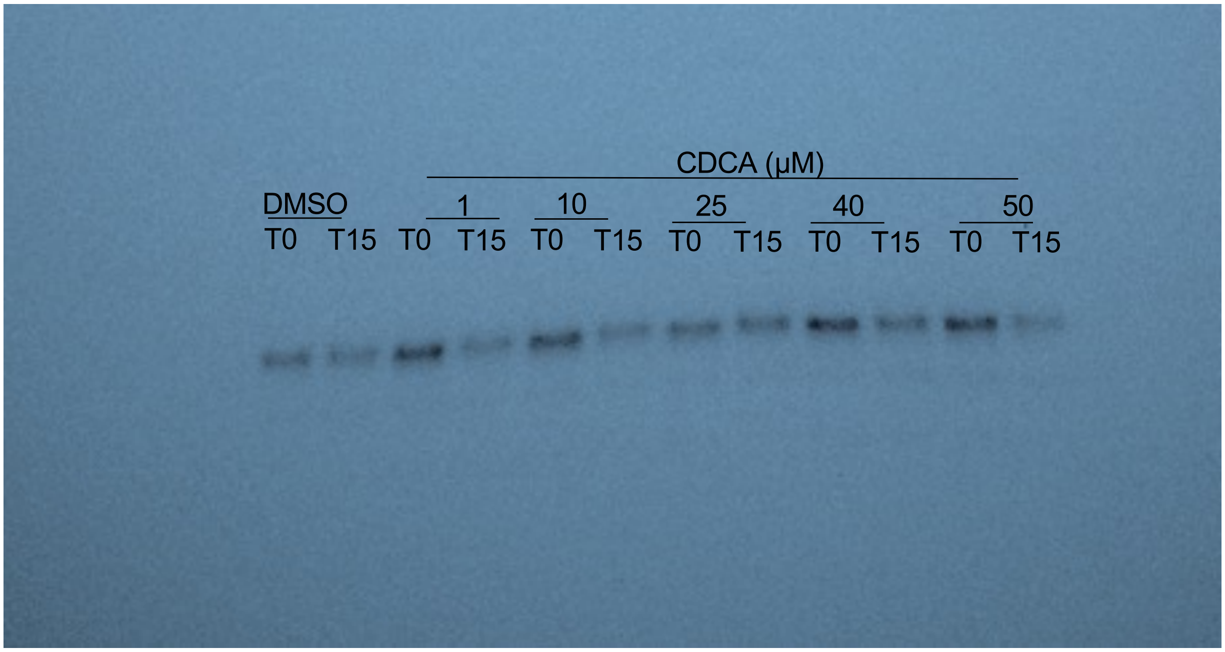

Full un-edited gels for Supplemental Figure 1C

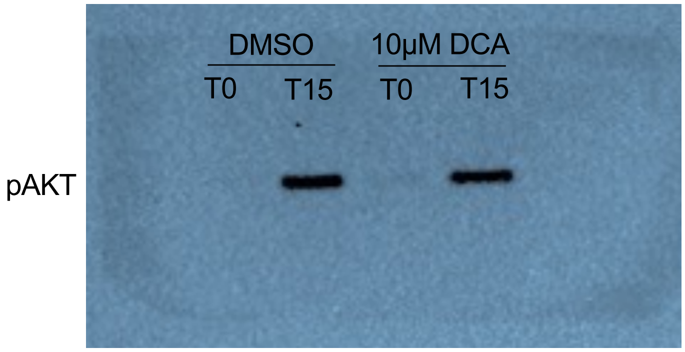

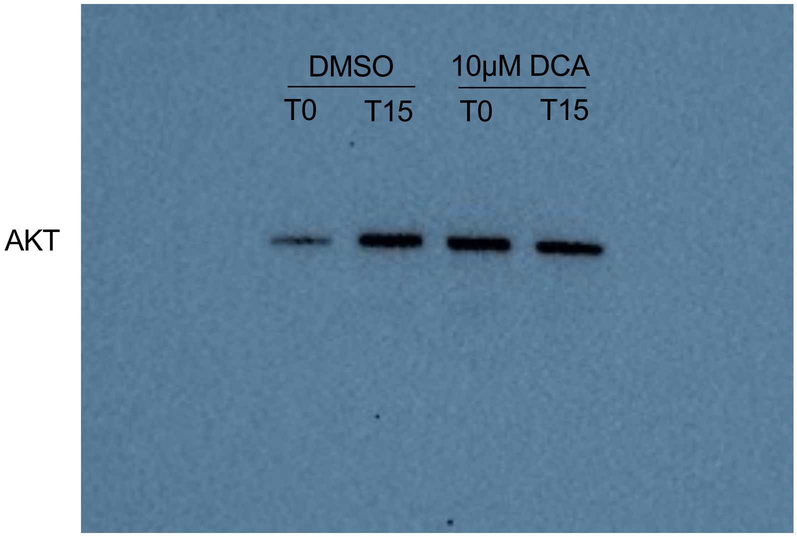

Supplement: Supplemental data [file jci-132-152961-s071.pdf]
